# Long-term memory of experienced jays facilitates problem-solving by naïve group members in the wild

**DOI:** 10.1038/s41598-023-46666-z

**Published:** 2023-12-07

**Authors:** Hyein Jo, Kelsey B. McCune, Piotr G. Jablonski, Sang‑im Lee

**Affiliations:** 1 Laboratory of Behavioral Ecology and Evolution, School of Biological Sciences, Seoul National University, Seoul, South Korea; 2 Institute for Social, Behavioral and Economic Research, University of California Santa Barbara, Santa Barbara, CA USA; 3 College of Forestry, Wildlife and Environment, Auburn University, Auburn, AL USA; 4 Museum and Institute of Zoology, Polish Academy of Sciences, Warsaw, Poland; 5 Laboratory of Integrative Animal Ecology, Department of New Biology, Daegu-Gyeongbuk Institute of Science and Technology, Daegu, South Korea

**Keywords:** Behavioural ecology, Long-term memory

## Abstract

Long-term memory affects animal fitness, especially in social species. In these species, the memory of group members facilitates the acquisition of novel foraging skills through social learning when naïve individuals observe and imitate the successful foraging behavior. Long-term memory and social learning also provide the framework for cultural behavior, a trait found in humans but very few other animal species. In birds, little is known about the duration of long-term memories for complex foraging skills, or the impact of long-term memory on group members. We tested whether wild jays remembered a complex foraging task more than 3 years after their initial experience and quantified the effect of this memory on naïve jay behavior. Experienced jays remembered how to solve the task and their behavior had significant positive effects on interactions by naïve group members at the task. This suggests that natural selection may favor long-term memory of solutions to foraging problems to facilitate the persistence of foraging skills that are specifically useful in the local environment in social birds with long lifespans and overlapping generations.

## Introduction

Many organisms from taxa across the animal kingdom rely on learning and memory for survival^1^. Learning facilitates repeated behavioral interactions with environmental factors for foraging, detecting and avoiding predators, and securing mating opportunities. Similarly, long-term memories are particularly likely to convey fitness benefits under seasonally changing environments where environmental factors predictably change over long timescales and so memories of previous successful interactions can still be useful^2^. However, long-term memory is energetically costly to create and maintain^3^, so the benefit of retaining these memories must outweigh the costs. For example, caching birds, like species belonging to the Corvidae and Paridae families, rely on seasonally available food that they cache for a later time when environmental conditions are more harsh and food is less abundant. The fitness consequences and cognitive mechanisms for the long-term memory of cache locations and food quality in these species have been thoroughly examined^4–6^ such that we understand how the benefit of long-term memory outweighs the cost. However, less is known about the evolutionary benefits of long-term memories in wild animals beyond the context of caching.

In comparison to studies of long-term memory for cached food, evidence for long-term memory in other contexts is sparse. Captive great apes from two separate studies were trained in tool-use tasks and could selectively retrieve relevant memories after more than 3 years without practice^7,8^. In the wild, primates can also remember complex foraging tasks after more than 2 years and knowledgeable individuals affect the behavior of naïve individuals on the task^9^. Furthermore, wild frog-eating bats retained memories of a novel acoustic cue indicating the presence of prey for up to 4 years without reinforcement^10^. In birds, one study found that wild American crows were able to discriminate between threatening and non-threatening people after more than 2 years^11^. Whereas in captivity, ravens recognized threatening people for up to 4 years^12^ and jungle crows also remembered a color discrimination task for up to 10 months^13^. These studies show that long-term memory across contexts is possible in species in both the wild and in captivity. However, further research is needed to understand potential evolutionary benefits and functions of long-term memory for foraging skills across taxa.

Long-term memory may be particularly beneficial in social species. In addition to solving foraging problems independently, individuals that live in social groups have the opportunity to learn from others that may remember solutions to foraging tasks^14^. When species are capable of both long-term memory and social learning, it is possible for persistent behavioral traditions to occur within social groups (i.e., “culture”, defined as the repeated transmission of novel behaviors over time through social learning^15^). For example, chimpanzees and New Caledonian crows can create tools to solve novel foraging problems in their environment and then naïve individuals adopt this behavior through social learning^16,17^. In some cases, the memories of older group members can impact the survival and fitness of the social group. For example, the removal of matriarch elephants, the most experienced member of the herd, can negatively affect the whole herd through reduced fitness due to loss of vital knowledge such as the location of water holes and appropriate responses to different types of predators^18^. Consequently, it is becoming more obvious that it is important to evaluate which species, and in what conditions, long-term memory is needed for social learning and group persistence^19^.

**Figure 1:**
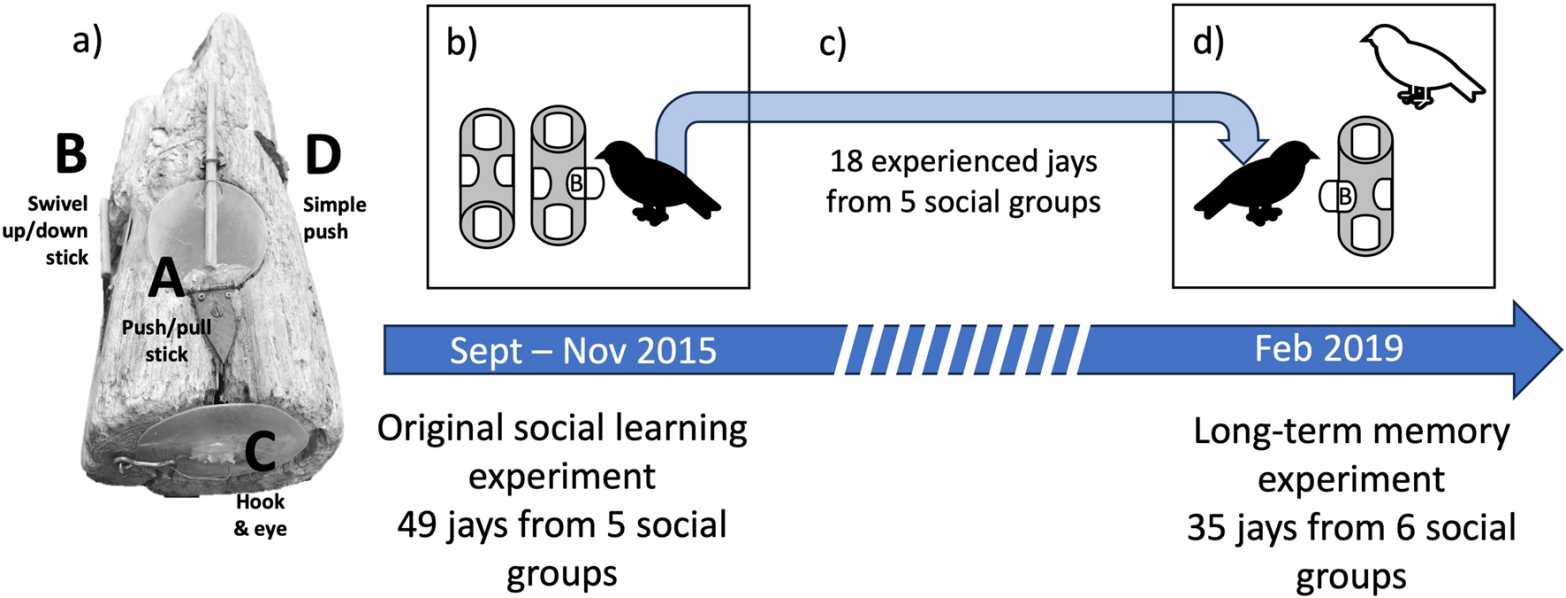
Illustration of the experimental design showing (**a**) the apparatus for the foraging task with the doors closed and locked, as during experimental trials. There are 4 food-holding compartments covered by doors with locks on 3 of the 4 doors (complex doors A, B, and C) that open in different ways. The fourth door (simple door D) on the right side pushes in to allow access to the compartment. (**b**) McCune et al. 2022 used this apparatus in 2015 to determine which social learning mechanisms jays use in this population, which resulted in 49 participating jays (bird in black) that learned how to open at least one of the apparatus doors (B door in this illustration). (**c**) No jays saw the apparatus again until 2019 and 18 jays that had learned to open the apparatus doors in 2015 persisted in the population across this time interval. (**d**) In the long-term memory experiment, we tested whether these experienced jays (in black) remembered how to interact with the apparatus and quantified the effect of their behavior on naïve jays (in white) that were not present for the 2015 experiment.

In this study, we tested for long-term memory of a complex problem-solving task and evaluated the impact of this memory on naïve individuals. Our study system was the Mexican jay (*Aphelocoma wollweberi*), a group-living, cooperative species in the family Corvidae. We compared the performance of free-living Mexican jays at an extractive multi-door foraging task (Fig. 1a) among individuals that differed in whether they had experience with the identical task from an experiment several years prior^20^. We hypothesized that Mexican jays have the capacity for long-term memory and that, as a social species, long-term memory may benefit naïve group members. We predicted that jays that had previously learned how to interact with the foraging task (“experienced jays”) would more frequently attempt and solve the four options (“doors”) on the task than the jays that were not present during the original study (“naïve jays”; P1). In addition, the doors on the foraging task varied in complexity which could affect whether naïve individuals are able to replicate the behavior of experienced group members, or the ability of an experienced jay to successfully reproduce a previously learned behavior^21^. Therefore, we further predicted that performance at the task and the effect of experience would vary based on door complexity, where jays would exhibit more attempts and solves at the simple door than the complex doors (P2). Lastly, interactions with the foraging task by experienced jays may affect the behavior of naïve jays, and we predicted a temporal relationship whereby naïve jays would attempt and solve more at the foraging task after observing experienced jays (P3; Table 1).

**Table 1:** Model output values from our generalized linear mixed model analyses testing P1 and P2, as well as the Cox proportional hazards model output for testing P3, for both the number of solves and the number of attempts. Sample sizes for each prediction are listed in parentheses in the first column. In the model structure detailed in column 2, the reference factor level is listed first. Not shown in the P1 & P2 model structure is a model offset for the number of doors of each type (complex = 3 doors, simple = 1 door) to account for unequal availability. “HR” stands for hazard ratio − the difference in the latency to attempt or solve after observing group member interactions (HR = 1 indicates no difference).

Prediction	Model	Estimate	Estimate 95% CI	std. error	*p*-value
P1: Experienced jays will attempt and solve more than naïve jays P2: Jays will exhibit more attempts and solves at the simple door than the complex door (n = 35)	1. Attempts ~ Naïve/experienced + Complex/simple + Observations + (1|Group) + (1|ID)	0.90 − 0.36 0.93	− 0.37–2.17 − 1.44–0.72 0.33–1.55	0.65 0.29 0.31	0.16 0.21 **0.002**
	2. Solves ~ Naïve/experienced + Complex/simple + Observations + Complex/simple*Naïve/experienced + Complex/simple*Observations + (1|Group) + (1|ID)	2.14 3.38 0.14 − 1.34 0.88	0.69–3.59 2.07–4.69 − 0.45–0.73 − 2.75–0.07 0.39–1.37	0.74 0.67 0.30 0.72 0.25	**0.004** ** < 0.001** 0.64 0.06 ** < 0.001**
P3: Naïve jays will attempt and solve more after observing experienced jays (n = 17)	3. Latency to Attempt ~ Observations + (1|Group)	− 1.64 (HR = 0.19)	− 3.90–0.62 (0.02–1.86)	1.15	0.15
	4. Latency to Solve ~ Observations + (1|Group)	0.60 (HR = 1.82)	0.07–1.13 (1.07–3.09)	0.27	**0.03**

Significant values are in bold.

## Results

A total of 35 jays from 6 social groups participated in this experiment by being present to observe group mates’ interactions at the multi-door foraging task, or to interact directly with the task. Of these 35 individuals, 18 were considered “experienced” because they had either been explicitly trained on complex door opening techniques for the original social learning experiment in 2015 (3 individuals) or had learned to solve the task through participation in the social learning experimental trials in 2015. The remaining 17 of the 35 jays were considered “naïve” as they had no experience with the foraging task, either because they were not present in the population in 2015 or because they were part of a group that was not included in the social learning experiment in 2015.

We were unable to determine the sex composition and exact age for jays in this population. This species is not sexually dimorphic and while only females incubate the eggs, only a few individuals breed in each group each year and we conducted our experiments in the non-breeding season. However, juveniles (ages 1–3) are distinguishable by light coloration on the bill, so we were able to provide rough estimates of the age of the color banded jays. During the 2019 experiment reported here, 4 of the 17 naïve jays were juveniles. These data were too imprecise to include as a covariate in our models, but we do not believe age had a large impact on performance because our results were the same if we dropped these 4 juveniles from our analyses (see supplementary information).

We found evidence that the experienced jays had retained the memory of how to solve (open the doors to retrieve the food item) the foraging task. Two of the three jays trained on lock-opening techniques for the social learning experiment in 2015 were each able to solve these complex doors on the apparatus less than 4 min after the start of the first trial in 2019, which was significantly faster than their performance in 2015 (see supplementary information). One of these previously trained jays solved the task 24 times, while another solved it 5 times. The third jay that had been trained to open a complex door in 2015 did not interact directly with the task in 2019. Of the other 15 experienced jays, 11 (73%) solved at least one door on the foraging task and it was always a door type that they had experience solving from the 2015 experiment (see supplementary information). In contrast, 8 of the 17 naïve jays (47%) solved at least one door. The simple door, which did not have a lock, was solved by 61% of the experienced jays and 41% of the naive jays. The 3 complex doors were collectively solved by 28% of the experienced jays and 18% of the naïve jays. Because of our short experimental duration and the complex behaviors required to access food from the locked doors, we also considered the number of attempts (touched the apparatus with the foot or bill but failed to obtain a food item) jays made as an indicator of the motivation to interact with the task. 72% of experienced jays and 41% of naïve jays attempted at a door on the foraging task.

**Figure 2:**
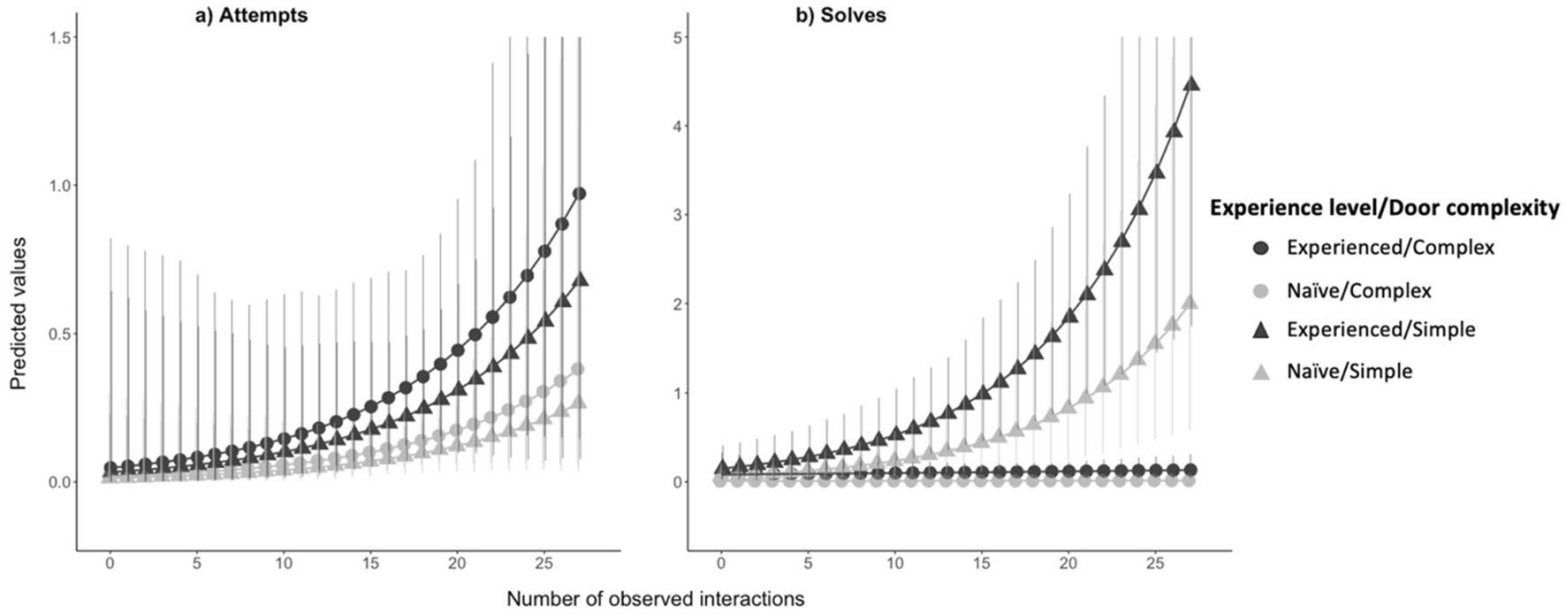
Plots of the predicted performance (points) and 95% confidence intervals (vertical lines) from our two models testing P1 and P2. (**a**) Predicted number of attempts increased for both experienced (black) and naïve (light gray) jays on the complex (circles) and simple (triangle) doors after observing interactions by conspecifics. (**b**) In the absence of observed interactions, predicted number of solves by jays at the foraging task varied by experience as predicted in P1, where experienced jays (black) solved more than naïve jays (gray) at the complex doors (circles). Observing group members interact led to more solves of the simple door (triangles) by both naïve and experienced jays.

We analyzed whether experienced jays, that were present and interacted with the apparatus during the 2015 experiment, were more successful on our foraging task than naïve jays (P1) and how the complexity of the door (i.e., had a lock or not) affected performance (P2). We first quantified performance at the foraging task in terms of the number of *attempts* jays made at the different doors during trials. There was no evidence that interaction terms improved the fit of the model to these data (likelihood-ratio test *p-values* > 0.05 for all nested model comparisons) so we interpreted the simplest model that included only the main effects for experience and door complexity. In addition, we included a covariate for the number of interactions (attempts and solves) by other birds that a focal jay observed to control for the social learning opportunities that may occur during trials (“observations”), as well as random terms for social group and individual ID to account for repeated measures (Table 1: Model 1). We found that the difference between naïve (n = 17; mean ± se = 0.88 ± 0.26) and experienced jays (n = 18; 1.68 ± 0.36) in the number of attempts they made at the foraging task was not statistically significant (ß = 0.90, *p* = 0.16; Fig. 2A). However, for all jays there was a significant effect of observing group members interact at the task (*ß* = 0.93, *p* = 0.002), where jays that observed more interactions by group members made more attempts at the task. We found no support for our second prediction (P2) that attempts would vary based on whether the door that jays interacted with was simple or complex. There was no statistically significant difference in the number of attempts to the simple door (0.37 ± 0.16) relative to the complex doors (1.6 ± 0.29; *ß* =  − 0.36, *p* = 0.21; Fig. 2A).

We then evaluated performance in terms of the number of *solves* by each jay. We found that a model that incorporated two 2-way interaction terms between experience and door complexity, as well as door complexity and observations (Table 1: Model 2) significantly improved the fit of the model to our data over a model that only included main effects (χ^2^ = 4.03, *p* = 0.04). To interpret the main effects while accounting for the interaction terms, we used pairwise post-hoc tests with a Tukey *p*-value adjustment in the package “emmeans”^22^. In support of P1, we found that, when jays have not observed any interactions by group members, experienced jays made more solves compared to naïve jays at the complex doors (*ß* =  − 2.14, *p* < 0.001) but not the simple doors (*ß* =  − 0.80, *p* = 0.47). Thus, the effect of door complexity on the initial number of solves was opposite to our second prediction. However, the interaction between door complexity and observations was statistically significant (*ß* = 0.88, *p* < 0.001), indicating that observing more interactions by group members at the task related to an increase in solves specifically at the simple door compared to the complex doors (Fig. 2B).

**Figure 3:**
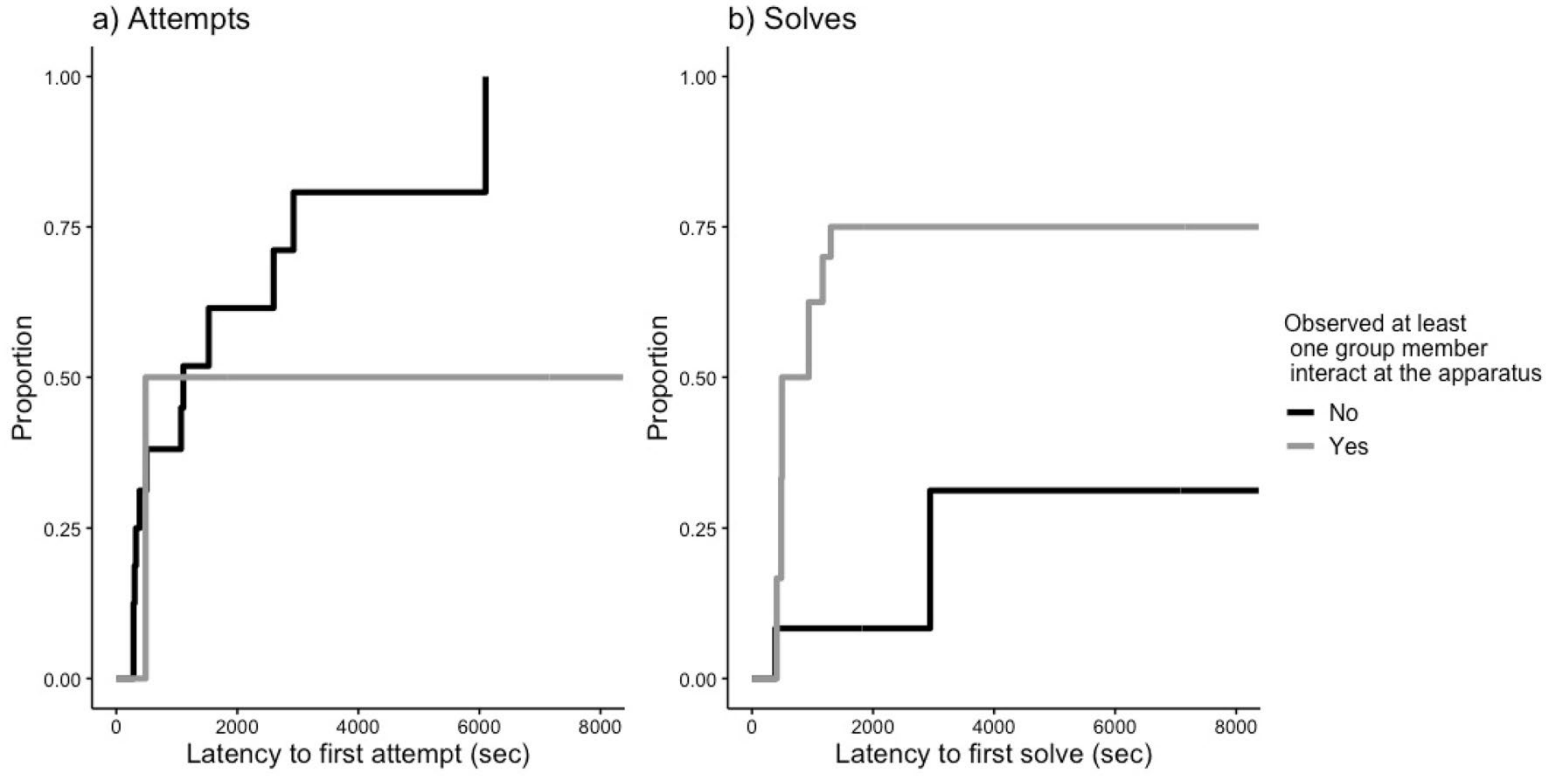
Survival curves illustrating the proportion of naïve jays at each time step that made their first attempt or solve at the foraging apparatus. The black and gray lines compare the latency of naïve jays to (**a**) attempt and (**b**) solve the foraging apparatus based on whether they obtained any social information (observations of group members interacting with the apparatus) prior to their first attempt or solve. In black are the naïve jays that did not observe any group members interact with the task and in gray are naïve jays that observed at least one group member interact.

Our last prediction (P3) was that the presence of jays interacting at the foraging apparatus may lead to an increase in the interactions by naïve jays. We evaluated the effect of observing group members interact with the task on the latency of naïve jays to first attempt (Table 1: Model 3) or solve (Table 1: Model 4) any door. We found that observations had no effect on the likelihood that naïve jays attempted a door for the first time (Hazard ratio = 0.19, *p* = 0.15; Fig. 3A). Conversely, observations increased the probability that a naive jay will solve a door for the first time by 82% (Hazard ratio = 1.82, *p* = 0.03; Fig. 3B).

## Discussion

We found that Mexican jays can remember how to solve a complex foraging task after nearly 4 years, and this resulted in experienced jays solving more at the complex doors on the foraging task than naïve jays when jays had not observed any interactions by group members at the task. However, observing group member interactions had a significant effect on performance of all individuals at the task, and specifically increased the number of solves of the simple door. Observing group members interact also significantly decreased the latency to solve any door by naïve jays. Overall, the synergistic effects of the long-term memory of the experienced jays and social information use by the naïve jays increased the likelihood that more members of the group could benefit from this novel foraging resource.

By increasing our understanding of animal cognitive traits and the evolutionary benefits of these traits, we can better predict the potential ability of animals to adapt to the changing world. Much of corvid foraging ecology revolves around caching and recovering food items, which depends on spatial memory. While it is important to know the mechanisms and duration of spatial memory, long-term memory related to other aspects of foraging ecology is also worthy of study. For example, as humans continue to alter the natural world, it is likely that species will encounter more novel foraging problems (e.g., keas opening trash cans;^23^). Consequently, for predicting species and population persistence in human-modified environments it is important to assess the memory retention and social transmission of solutions to novel foraging problems. The long duration of memory retention that we show (nearly 4 years) extends the known limit of avian memory. Brain mass is energetically costly to produce and maintain^3^, so it is likely that long-term memories for solutions to novel foraging problems increase survival or fitness of individuals in this population in some way. We demonstrate that memory retention functions to increase the foraging success of experienced individuals, as well as positively influencing the foraging success of naïve individuals. Together, these traits could facilitate the transmission of new behaviors through populations to increase the ability of individuals to utilize novel resources in human-modified environments (e.g., the opening of milk bottles by birds;^24^).

In this way, knowledge of the content and function of long-term memories could facilitate conservation of threatened and endangered species. Through training in alternative or novel foraging resources, or in novel predator avoidance, conservation practices could take advantage of memory capacity for increasing species survival and fitness. For example, North Island robins (toutouwai) that learned to solve a foraging task remembered how to solve the task after more than a year^25^. The majority of individuals in this species live in protected reserves with exclosure fences to prevent predation by introduced predators. However, this long-term retention of information could lead to increased survival and fitness in trained individuals that disperse beyond the reserve^26^. This technique could be particularly useful for species that live in social groups and are able to socially learn optimal foraging choices for particular environments. By training one or a few members of the social group, the information can be transmitted to the rest of the group without managers needing to train each individual^27,28^.

The evidence for the cognitive traits we focused on here could indicate the capacity in this system for other, more cognitively demanding abilities. While we found evidence for social learning by naïve jays, they did not exactly replicate the experienced jay behavior because naïve jays were less likely to solve the complex door compared to experienced jays. This is similar to the results of our previous examination of social learning mechanisms in this species^20^ where we also found that naïve jays did not copy the lock-opening methods of the trained demonstrators. Instead, in both experiments we found that the presence of experienced jays interacting with any door on the apparatus led to increased interactions by naïve jays. The coexistence of social learning and long-term memory for solving complex foraging problems is interesting because these traits are important prerequisites for the evolution of certain animal cultural traditions^15^. For example, the use and manufacture of tools by New Caledonian crows is thought to represent cultural behavior because efficient tool manufacture and use requires that naïve individuals socially learn from knowledgeable adults^29,30^, and the form of the tool shows regional differentiation^31^. However, because naïve jays do not exactly copy the behavior of experienced individuals, further research is needed to determine whether social learning and long-term memory have led to different Mexican jay social groups exhibiting any candidate cultural behaviors. Additionally, the long-term memory retention observed in the Mexican jay may indicate the potential for episodic-like memory in this species. Dally and colleagues^32^ found that captive California scrub jays, a closely related species, are capable of episodic-like memory, defined as retaining what-where-when information^33^. In our study, the Mexican jays were capable of remembering the “what” and “where” component of a complex problem-solving task, indicating that Mexican jays may be a good study system for testing episodic-like memory in the wild.

We designed our study to determine whether individuals that previously learned to open complex locks would immediately remember this skill several years later, without re-learning the task through trial-and-error. Additionally, we aimed to determine whether the presence of experienced jays would quickly affect the interactions of naïve jays at the novel task. Thus, our design included short sampling intervals (few trials, short trial durations) that might have reduced the detection of some of the learning by naïve jays that may happen over longer timescales. Despite this, the opportunity to observe group members interact with the foraging task had a significant impact on attempts and solves of both experienced and naïve jays. The variation in the responses of jays to this foraging task would be interesting to further investigate in future studies with larger sample sizes or longer sampling intervals. For example, future analysis could add to our results by examining other factors that might relate to the variation in naïve jays’ solving performance by describing whether variation in personality traits affects whether experienced jays remember a previously learned skill or how quickly naïve jays approach and learn the complex task. To better understand when the evolution of long-term memory is favored, additional studies comparing long-term memory of non-caching species would be useful for testing whether the presence of long-term memory of foraging problems in birds is linked with caching behavior.

Long-term memory likely works through diverse mechanisms according to species traits, evolutionary history, and the species-specific ecological niche. Although it can be financially and logistically difficult to study the same individuals across long time spans, especially in the wild, we believe it is a worthwhile endeavor for several reasons. With more information on the mechanisms and expression of long-term memory in wild animals, we may be able to better develop management plans for social species with long-term memory that may show cultural traditions in behavior (e.g., whales;^34^). Furthermore, studying long-term memory in non-human animals can help elucidate the factors contributing to the evolution of human cognitive capacity and memory retention^15^. Lastly, a better understanding of long-term memory in different animal models could be applied to technologies such as artificial intelligence (e.g., Animal-AI Testbed, an artificial intelligence evaluation platform derived from animal cognition;^35^), thus leading to interdisciplinary convergence of different fields and further expansion of the function and consequences of long-term memory.

## Methods

### Study site and subjects

We conducted this study around the American Museum of Natural History’s Southwestern Research Station in Portal, AZ in the winter of 2019. Subjects for the experiment involved individually color-banded Mexican jays from six social groups (flocks). A similar experiment that took place in 2015, and is described in McCune et al.^20^, occurred in the same location with the same flocks, though there was some turnover in individuals (Fig. 1). All methods were approved by the Southwestern Research Station, USGS bird banding lab permit #23,211, Arizona Game and Fish permit #SP646020 & the AMNH Animal Care and Use Committee. We followed all guidelines for ethical animal research outlined in the Association for the Study of Animal Behaviour Guidelines for the Treatment of Animals in Behavioural Research and Teaching. We had landowner permission to do research at all sites where flocks occurred.

### Foraging apparatus

To measure problem-solving in this experiment, we used a foraging apparatus that was a log with four compartments, each compartment was covered by a transparent door that opened in a different way. One door could be opened simply by pushing it in, while the other three doors were additionally secured with distinct locks: a stick that could be swiveled up or down over the door, a stick that could be pushed or pulled over the door, and a hook-and-eye style lock (Fig. 1a). All jays in this population were trained to come to a whistle for food which insured that most individuals arrived at the testing location as soon as we put out the apparatus and whistled. After a door was opened during a trial, the experimenter would slowly approach to refill the food items, then close and lock the door. Jays were habituated to the experimenter’s presence and always returned to the apparatus after the experimenter retreated.

### Summary of the similar, previous experiment

We conducted a social learning experiment in 2015^20^ to determine which social learning mechanisms are used by jays from two species, one of which (the Mexican jay) is the focus here. We trained two adult Mexican jays from 3 out of 5 flocks as demonstrators on one of the lock-opening methods on the foraging apparatus. To isolate two individuals from the group, demonstrators were trained in captivity in large aviaries on the campus of the Southwestern Research Station for a maximum of three weeks. We first habituated the captive jays to the apparatus until they recognized it as a food source and then began training on the opening methods for either the top (push–pull stick) or left-side (swivel stick) door locks. After demonstrator jays were consistently and efficiently opening the lock, we released them back into their flock. We then conducted social learning trials in the center of each flock’s territory. Trials were conducted in the morning and were a maximum of 2 h long, or until 30 min had passed where no jays had touched the apparatus. Each flock received between 9 and 16 trials, in which naïve jays learned how to open the doors on the foraging apparatus.

### Long-term learning experiment

In 2019, we returned to the Southwestern Research Station with the same foraging apparatus for the current experiment, described here, that included jays that were present for the previous experiment (n = 18), as well as naïve jays that were new to each flock since 2015 (n = 17). Before beginning experimental trials, we habituated all jays to the apparatus as a non-threatening source of food by allowing individuals to eat peanuts and sunflower seeds from inside, on top, and around the apparatus. During habituation, the doors were secured in the open position so that no learning about door opening techniques could occur at this stage. Habituation occurred on each territory until the majority of jays in each flock were seen eating from the apparatus. No flock required more than 2 days of habituation. We then put the closed and locked foraging apparatus out in the center of each territory for between two to four trials of 15 to 45 min for each of six flocks. We ended a trial after 45 min, or if no jay interacted with the apparatus for 20 min. All jays in each flock (range 6–9 jays per flock) were able to freely interact with the task during trials. All trials were video recorded such that the apparatus and 2 m of ground on each side were in the frame for detecting observing jays. The original experiment in 2015 found that jays outside of the 2 m radius potentially observing interactions at the apparatus did not contribute significantly to the results (McCune unpublished data). The videos were coded later by an observer naïve to the experimental design and hypotheses.

### Statistics and reproducibility

From the video recordings of experimental trials we determined the color-band combination of all individuals to come within 2 m of the foraging task. We recorded all interactions with the foraging apparatus as in the original social learning experiment in 2015^20^ to note the time of interaction, the individual’s identity, whether that individual learned to interact with the apparatus during the 2015 experiment or not (“experienced” or “naïve”), where on the foraging apparatus they interacted, whether a food item was successfully obtained, and the identities of all other jays in the frame. We considered that a jay had an “observed interaction” when it was within the video frame while another group member was interacting (attempting or solving) at the apparatus. We defined an “attempt” as touching the apparatus with the foot or bill but failing to obtain a food item. We defined a “solve” as manipulating a door and/or lock on the foraging apparatus in a way that permits the individual to get the food item from the compartment. Note that, as the simple door did not have a lock, it could be opened with one touch and therefore was counted as a solve with zero attempts.

To determine support for our first two predictions (P1, P2), we compared the number of attempts and the number of solves between “experienced” jays that had interacted with the foraging apparatus during the 2015 social learning experiment and “naïve” jays that had no prior experience with the foraging task. To quantify and control for the learning opportunities that may occur during the experiment, we also included in all models a covariate for the total number of attempts and solves on the apparatus by other birds that a focal jay observed. We combined observations of group member attempts and solves in this variable because social learning can occur even when naïve individuals do not see the experienced individuals acquire a reward^14^. We included an additional explanatory variable for door complexity. The foraging apparatus contained 3 complex doors and 1 simple door, so to account for this differential availability our model included an offset term consisting of the number of doors in each complexity category to model the proportion of attempts or solves to complex and simple doors. Lastly, we included a random effect for flock ID because there were multiple jays in the sample from the same social group, and a random effect for individual ID because there were multiple values for each individual (one value for each of the four doors).

Our response variables for these models were counts, and we verified that the distribution of the data met the assumptions for Poisson models. The effects of observations or door complexity could affect the number of solves or attempts of knowledgeable and naïve jays differently. So, in each model we additionally tested for interactions among the explanatory variables. To avoid overfitting the models, we used likelihood ratio tests to evaluate whether individual interaction terms and combinations of interaction terms significantly added to the variance explained by the model. Note that we additionally ran identical models where the response variable was a rate (attempts or solves per trial time), but results were qualitatively identical to the count models that we present here (see supplementary information).

For P3 we further investigated the effect of the long-term memory of experienced jays on the ability of naïve jays to socially learn about this novel foraging task. Subsetting the data to just the performance of naïve individuals (n = 17), we analyzed the time to first attempt and time to first solve using Cox proportional hazard survival models with a time-dependent covariate^36^ of observations, as well as a random effect for flock ID. Survival analyses are appropriate because some naïve individuals never solved any door on the foraging task within the experimental time frame and these censored values are statistically accounted for^37^. We verified that both the attempts and solves survival models met the assumption of proportional hazards.

We used RStudio for all data analyses and visualization, specifically the R function “glmer” in the lme4 package^38^, coxme^39^ and ggplot2^40^. All data and code used in this study are available in the KNB public repository (https://knb.ecoinformatics.org/view/doi:10.5063/F1WD3Z1T).

### Supplementary Information

Supplementary Information.

## Data Availability

All data and code used in this manuscript are available in the KNB data repository at https://knb.ecoinformatics.org/view/doi:10.5063/F1TM78KP.
