# Supplementary material for: Long-term memory of experienced jays facilitates problem-solving by naïve group members in the wild
Source: Sci Rep. 2023 Dec 7;13:21593. doi: 10.1038/s41598-023-46666-z (PMC10703848; doi:10.1038/s41598-023-46666-z)
Supplement: Supplementary file 1 — Supplementary Information. [file 41598_2023_46666_MOESM1_ESM.pdf]

Supplementary materials for *Long-term memory of experienced jays facilitates problem-solving by naïve group members in the wild*

2023-08-31

Background

This file contains code for analyses presented in our manuscript "Long-term memory of experienced jays facilitates problem-solving by naïve group members in the wild". Data to run these analyses are available in the KNB public repository (https://knb.ecoinformatics.org/view/doi:10.5063/F1WD3Z1T). This is the second revision of this manuscript, and we include analyses here for addressing a few of the reviewer comments. Also included are supplemental analyses and figures.

Our goal was to evaluate whether jays that experienced a social learning task in 2015 using the same foraging apparatus would still remember how to solve the task when presented with it again in 2019. Secondly, we wanted to quantify the effect of this long-term memory of experienced jays on the solving performance of naïve jays that had never seen this foraging problem before.

Experience categories:

P1/P2 - How are interactions with the apparatus related to experience, door complexity and observations of group member interactions - Experienced jays (present for original experiment in 2015) n = 18 - Naive jays (not present for original experiment in 2015) n = 17

P3 - For the 17 Naive jays, how does observing group members interact influence the latency to interact

Variables:

attempt - a touch to the apparatus with the foot or bill, but failing to obtain a food item

solve - manipulating a door and lock on the foraging apparatus in a way that permits the individuall to get the food item from the compartment

observations - number of attempts and/or solves the focal jay sees a group member make at the foraging apparatus

experienced - whether the jay was present for the original social learning experiment in 2015 and so gained experience opening doors on the foraging apparatus ("Yes"), or whether the jay had never before seen the foraging apparatus ("No")

complexity - describes how difficult the doors on the foraging apparatus are to open. A, B, & C doors have locks and are "complex" whereas D door does not have a lock and is "simple"

offset - a vector identifying the number of doors of each complexity type (3 complex doors, 1 simple door) that is included as an offset in the P1 & P2 Poisson models to account for the different availability of doors on the number of attempts and solves to those door complexity types

```r
sum.data19.o = read.xls("Rev1_MEJA 2019 long-term memory.xlsx",
                        sheet = "Summary Results")
data19.o = read.xls("Rev1_MEJA 2019 long-term memory.xlsx",
                    sheet = "Data")
trial.times = read.xls("Rev1_MEJA 2019 long-term memory.xlsx",
                       sheet = "Video clip annotations")

# remove unknown jays and jays that were present in the group
# according to an annual census but did not participate.
# During revisions we also removed jays from the GA group from the Excel file
# because only 1 banded jay participated with the foraging task
# from that group (22% of group)
sum.data19 = sum.data19.o[-which(sum.data19.o$Experienced=="UNK" |
                                   sum.data19.o$Participation.2019=="No"),-c(13:14)]
#n = 35

# create a column of total solves
sum.data19$sum.solves = sum.data19$NumberSolvesA + sum.data19$NumberSolvesB +
  sum.data19$NumberSolvesC + sum.data19$NumberSolvesD

# add trial times to sum.data19
sum.trial.times = aggregate(Clip.duration.sec.~Group, data = trial.times, FUN = "sum")
#add up all trial times for each group

sum.data19 = merge(sum.data19, sum.trial.times, by = "Group", all =T)
# add sum trial times to sum.data19 data frame
colnames(sum.data19)[14] = "sumTime" # change column name to be something shorter

# create a column of total observed interactions
# first create a data frame with observed interactions
obs = data19.o[,c(9:13)] #Behav, ID, Result, SawSuccess, SawInteraction
obs$count = 1

# we only want the Behav's related to observing interactions at a door
# (i.e. not the jay's own success or attempt)
obs = obs[-which(obs$Behav == "A" | obs$Behav == "AattemptD" | obs$Behav == "AattemptL" |
                   obs$Behav == "Ajiggle" | obs$Behav == "Alog" |
                   obs$Behav == "Ascrounge" | obs$Behav == "B" |
                   obs$Behav == "BattemptD" | obs$Behav == "BattemptL" |
                   obs$Behav == "BdownT" | obs$Behav == "BdownG" |
                   obs$Behav == "Bjiggle" | obs$Behav == "Bscrounge" |
                   obs$Behav == "BupG" | obs$Behav == "BupT" |
                   obs$Behav == "CattemptD" | obs$Behav == "CattemptL" |
                   obs$Behav == "Cscrounge" | obs$Behav == "Dattempt" |
                   obs$Behav == "Dpush"),]

unique(obs$Behav) #check the behavior categories are correct
```

```
##  [1] "ODpush"     "OAattemptD" "OBattemptL" "OCattemptD" "OBattemptD"
##  [6] "OBupG"      "OAattemptL" "OAjiggle"   "OA"         "ODattempt"
## [11] "OCattemptL" "OAlog"      "OBupT"      "OBdownT"    "OB"
## [16] "OBjiggle"
```

```
int = aggregate(SawInteraction ~ ID, data = obs, FUN = "sum")
succ = aggregate(SawSuccess ~ ID, data = obs, FUN = "sum")
obs.combined = merge(int, succ, by = "ID")
sum.data19 = merge(sum.data19, obs.combined, by = "ID", all = T)
sum.data19$SawInteraction[which(is.na(sum.data19$SawInteraction))]<-0
sum.data19$SawSuccess[which(is.na(sum.data19$SawSuccess))]<-0
#Also create a column for total observations
sum.data19$Observations = sum.data19$SawInteraction + sum.data19$SawSuccess


#Add in the number of attempts jays made at each door
att = data19.o[,c(9:13)]
att$count = 1
att = att[-which(att$Result == 1),] # Remove interactions that were successful.
# Remove observed interactions, because we now want attempts the focal jay made
att = att[-which(att$Behav == "ODpush" |att$Behav == "OAlog" |
                   att$Behav == "OBattemptD" | att$Behav == "OA" |
                   att$Behav == "OBattemptL" | att$Behav == "ODattempt" |
                   att$Behav == "OBupT" |att$Behav == "OBdownT" |
                   att$Behav == "OCattemptD" |att$Behav == "OAattemptD" |
                   att$Behav == "OAattemptL" |att$Behav == "OB" |
                   att$Behav == "OAjiggle" |att$Behav == "OBjiggle" |
                   att$Behav == "OBupG"| att$Behav == "OCattemptL"),]

unique(att$Behav) #check the behavior categories are correct
```

```
## [1] "AattemptD" "BattemptL" "CattemptD" "BattemptD" "AattemptL" "Dattempt"
## [7] "CattemptL"
```

```
attempts = aggregate(count ~ ID, data = att, FUN = "sum")
colnames(attempts)[2] = "sum.attempts"
sum.data19 = merge(attempts, sum.data19, by = "ID", all = T)
# Remove unbanded jays and jays that were present in group during census
# but then did not come near to interact with or observe interactions with the task.
sum.data19 = sum.data19[-which(sum.data19$ID == "UBA" | sum.data19$ID == "Y-X" |
                       sum.data19$ID == "BSS-KSX" | sum.data19$ID == "WVX-WVS" |
                       sum.data19$ID == "MKX-RMP" | sum.data19$ID == "RPP-OGX" |
                       sum.data19$ID == "S-X" | sum.data19$ID == "X-" |
                       sum.data19$ID == "RYO-GXR"),]

# Other jays have NAs if they made an attempt, solve or observation
# but not all 3. We will turn these values from NA to 0
sum.data19$sum.attempts[which(is.na(sum.data19$sum.attempts))]<-0
head(sum.data19)
```

```
##        ID sum.attempts Group NumberSolvesA NumberSolvesB NumberSolvesC
## 1    -PPX            0    KI             0             0             0
## 2 BGX-OVP           36    TK             7             2             0
## 3 BOX-KBS           13    HI             1             0             0
## 4  BX-SVG            6    UC             0             0             0
## 5 BXB-BSB            9    TK             0             5             0
## 6 BYP-OWX            0   XMO             0             0             0
##   NumberSolvesD Experienced Participation.2015 Participation.2019 Numeric.Age
```

```
## 1              0         Yes                  A                Yes             4
## 2              6         Yes                  D                Yes            5+
## 3              0          No                                   Yes            4+
## 4              0         Yes                A,C                Yes            4+
## 5              0         Yes              A,B,D                Yes            5+
## 6              0         Yes                  A                Yes            6+
##   Categorical.Age                         Notes sum.solves sumTime
## 1               A                                        0    4051
## 2               A Was YW-WBW re-banded 1/25/18          15    8372
## 3               A                Banded 1/28/18          1    6261
## 4               A                                        0    3113
## 5               A    Trained demonstrator on B           5    8372
## 6               A    Trained demonstrator on A           0    5034
##   SawInteraction SawSuccess Observations
## 1              0          1            1
## 2              4          4            8
## 3              7          1            8
## 4              0          0            0
## 5              4          4            8
## 6              1          1            2
```

Now we have a dataframe with the total number of attempts each jay made, as well as the number of times they observed group members interact with the apparatus.

P1/P2 analysis

Comparison of performance on complex doors and simple doors of experienced and naive jays.

- *Complex doors have locks = A, B, C. Simple door is unlocked = D.*
- *Experienced: Yes vs No*

```
# For the P1/P2 full model, we need a data frame that includes attempts and solves
# at each door so that we can compare attempts/solves by door complexity.
attxdoor = aggregate(count~ID + Behav, data = att, FUN = "sum")
# sum together the attempts at different parts of the doors (Behav) for each bird (ID)
attxdoor$Door = ifelse(attxdoor$Behav == "AattemptD" | attxdoor$Behav=="AattemptL",
                       "A",attxdoor$Behav)
# create a new column that labels the Door each Behav is directed at
attxdoor$Door = ifelse(attxdoor$Behav == "BattemptD" | attxdoor$Behav=="BattemptL",
                       "B",attxdoor$Door)
attxdoor$Door = ifelse(attxdoor$Behav == "CattemptD" | attxdoor$Behav=="CattemptL",
                       "C",attxdoor$Door)
attxdoor$Door = ifelse(attxdoor$Behav == "Dattempt", "D",attxdoor$Door)
attxdoor$complexity = ifelse(attxdoor$Door == "D","simple","complex")
# Create a column to identify the complexity of the doors listed in the "Door" column
colnames(attxdoor)[3] = "attempts"
attxdoor = aggregate(attempts ~ ID + complexity + Door, data = attxdoor, FUN = "sum")

succxdoor = sum.data19[,c(1,3:8,12,14:18)] # Now we need solves per door
# columns: ID, Group, NumSolvesA, NumSolvesB, NumSolvesC, NumSolvesD, Experienced,
# Categorical.Age, sumTime, SawInteraction, SawSuccess, Observations
# Experienced jays were present in the population to interact with or
# observe interactions with the task in 2015
```

```r
# only 2 experience jays only observed interactions in 2015, but then solved doors in 2019

colnames(succxdoor)[3:6] = c("A","B","C","D") #re-label columns
succxdoor = gather(succxdoor,Door,solves,c(A,B,C,D), factor_key = T)
# change the data sheet from "wide" format to "long" format
# Now, instead of each door having its own column, there is one column
# for door and a column for the number of solves per door.
succxdoor$complexity = ifelse(succxdoor$Door == "D","simple","complex")
# also create a door complexity column for solves

p12 = merge(attxdoor, succxdoor, by = c("ID","Door","complexity"),all = T)
# add together the solves and attempts data sheets
p12$attempts[which(is.na(p12$attempts))]<-0
# For jays that did not attempt on specific door types - replace the NA with a 0
p12$Experienced = as.factor(p12$Experienced)
# make this column a factor-type variable (rather than a character variable)
p12$complexity = as.factor(p12$complexity)
head(p12)
```

```
##        ID Door complexity attempts Group Experienced Categorical.Age sum.solves
## 1    -PPX    A    complex        0    KI         Yes               A          0
## 2    -PPX    B    complex        0    KI         Yes               A          0
## 3    -PPX    C    complex        0    KI         Yes               A          0
## 4    -PPX    D     simple        0    KI         Yes               A          0
## 5 BGX-OVP    A    complex       18    TK         Yes               A         15
## 6 BGX-OVP    B    complex       12    TK         Yes               A         15
##   sumTime SawInteraction SawSuccess Observations solves
## 1    4051              0          1            1      0
## 2    4051              0          1            1      0
## 3    4051              0          1            1      0
## 4    4051              0          1            1      0
## 5    8372              4          4            8      7
## 6    8372              4          4            8      2
```

This dataframe has the variables we need for our P1 & P2 models.

Round 1 Reviewer comment 53 - number of complex vs simple doors

Per reviewer comment 53, it is possible that there are more attempts or solves on complex relative to simple doors because there are 3 complex door and only 1 simple door. To account for this we can include an offset for the number of doors in each category for the poisson models.

```r
p12$offset = ifelse(p12$complexity == "complex",3,1)
```

This offset term is now included in all models below, and we reassessed which models now best fit the data.

P1/P2 SOLVES model selection

We used log-likelihood tests to evaluate which of multiple, hierarchical models best fit our data.

```
##        chisq        ratio          rdf            p
## 162.14183336   1.21001368 134.00000000   0.04930311
```

```
## Data: p12
## Models:
## m1: solves ~ complexity + Experienced + scale(Observations) + (1 | Group) + (1 | ID)
## m1int1: solves ~ complexity + scale(Observations) * Experienced + (1 | Group) + (1 | ID)
##        npar    AIC    BIC  logLik deviance Chisq Df Pr(>Chisq)
## m1        6 282.17 299.82 -135.09   270.17
## m1int1    7 284.17 304.76 -135.09   270.17     0  1     0.9968

## Data: p12
## Models:
## m1: solves ~ complexity + Experienced + scale(Observations) + (1 | Group) + (1 | ID)
## m1int2: solves ~ complexity * Experienced + scale(Observations) + (1 | Group) + (1 | ID)
##        npar    AIC    BIC  logLik deviance  Chisq Df Pr(>Chisq)
## m1        6 282.17 299.82 -135.09   270.17
## m1int2    7 280.72 301.31 -133.36   266.72 3.4504  1    0.06324 .
## ---
## Signif. codes:  0 '***' 0.001 '**' 0.01 '*' 0.05 '.' 0.1 ' ' 1

## Data: p12
## Models:
## m1: solves ~ complexity + Experienced + scale(Observations) + (1 | Group) + (1 | ID)
## m1int3: solves ~ Experienced + scale(Observations) * complexity + (1 | Group) + (1 | ID)
##        npar    AIC    BIC  logLik deviance  Chisq Df Pr(>Chisq)
## m1        6 282.17 299.82 -135.09   270.17
## m1int3    7 266.83 287.43 -126.42   252.84 17.335  1  3.133e-05 ***
## ---
## Signif. codes:  0 '***' 0.001 '**' 0.01 '*' 0.05 '.' 0.1 ' ' 1

## Data: p12
## Models:
## m1int3: solves ~ Experienced + scale(Observations) * complexity + (1 | Group) + (1 | ID)
## m1int12: solves ~ complexity * Experienced + scale(Observations) * Experienced + (1 | Group) + (1 | [illegible]
##         npar    AIC    BIC  logLik deviance Chisq Df Pr(>Chisq)
## m1int3     7 266.83 287.43 -126.42   252.84
## m1int12    8 282.72 306.25 -133.36   266.72     0  1          1

## Data: p12
## Models:
## m1int3: solves ~ Experienced + scale(Observations) * complexity + (1 | Group) + (1 | ID)
## m1int22: solves ~ complexity * scale(Observations) + scale(Observations) * Experienced + (1 | Group) [illegible]
##         npar    AIC    BIC  logLik deviance Chisq Df Pr(>Chisq)
## m1int3     7 266.83 287.43 -126.42   252.84
## m1int22    8 268.83 292.37 -126.42   252.84 5e-04  1      0.982

## Data: p12
## Models:
## m1int3: solves ~ Experienced + scale(Observations) * complexity + (1 | Group) + (1 | ID)
## m1int32: solves ~ complexity * scale(Observations) + complexity * Experienced + (1 | Group) + (1 | I[illegible]
##         npar    AIC    BIC  logLik deviance  Chisq Df Pr(>Chisq)
## m1int3     7 266.83 287.43 -126.42   252.84
## m1int32    8 264.81 288.34 -124.40   248.81 4.0267  1    0.04479 *
## ---
## Signif. codes:  0 '***' 0.001 '**' 0.01 '*' 0.05 '.' 0.1 ' ' 1
```

```
## Data: p12
## Models:
## m1int32: solves ~ complexity * scale(Observations) + complexity * Experienced + (1 | Group) + (1 | II
## m1int42: solves ~ complexity * Experienced + scale(Observations) * Experienced + complexity * scale(
##         npar    AIC    BIC  logLik deviance  Chisq Df Pr(>Chisq)
## m1int32    8 264.81 288.34 -124.40   248.81
## m1int42    9 266.65 293.13 -124.33   248.65 0.1558  1      0.693
```

```
## Data: p12
## Models:
## m1int32: solves ~ complexity * scale(Observations) + complexity * Experienced + (1 | Group) + (1 | II
## m1int3w: solves ~ complexity * Experienced * scale(Observations) + (1 | Group) + (1 | ID)
##         npar    AIC    BIC  logLik deviance  Chisq Df Pr(>Chisq)
## m1int32    8 264.81 288.34 -124.40   248.81
## m1int3w   10 265.47 294.88 -122.73   245.47 3.3405  2     0.1882
```

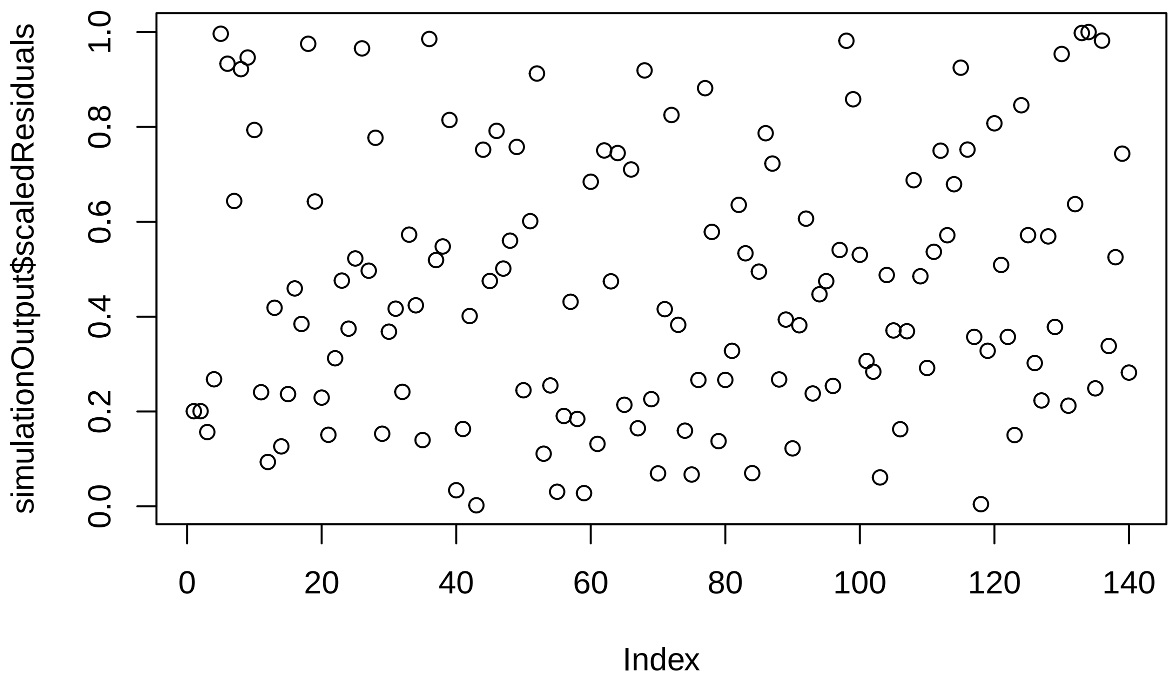

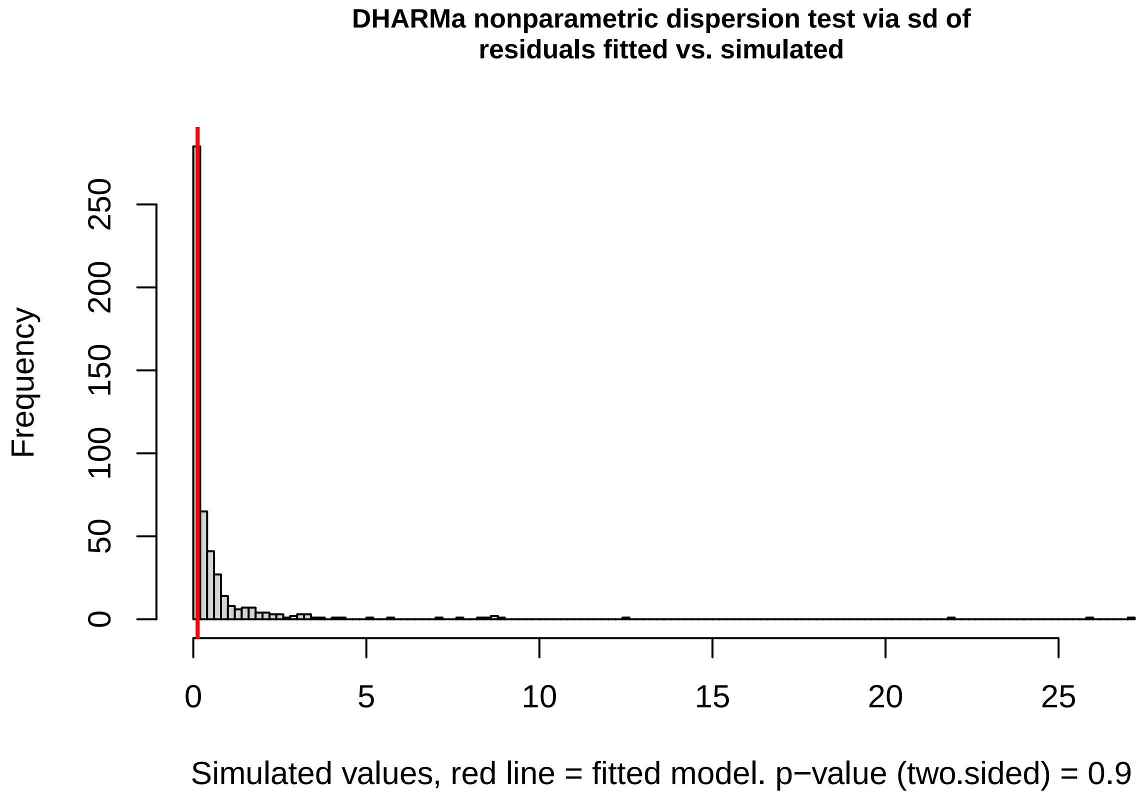


```
##
##  DHARMa nonparametric dispersion test via sd of residuals fitted vs.
##  simulated
##
## data:  simulationOutput
## dispersion = 0.16998, p-value = 0.9
## alternative hypothesis: two.sided
```

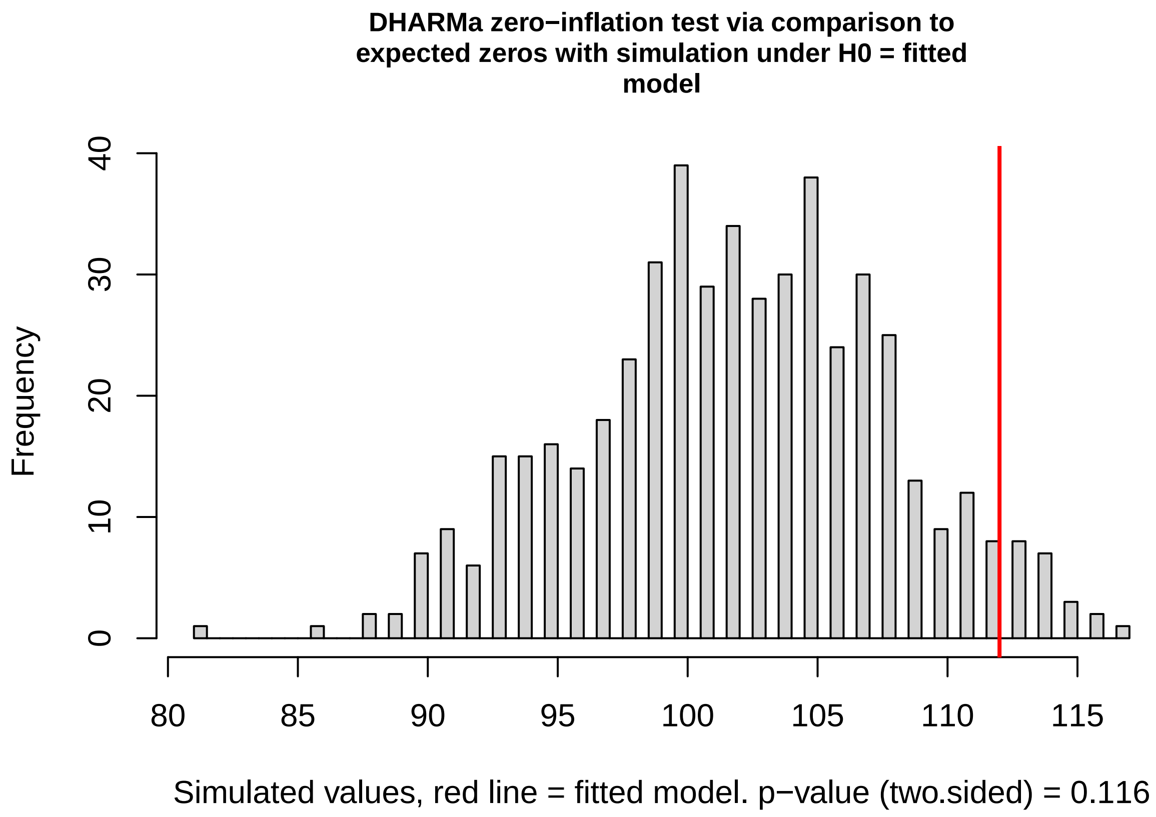


```
##
##  DHARMa zero-inflation test via comparison to expected zeros with
##  simulation under H0 = fitted model
##
## data:  simulationOutput
## ratioObsSim = 1.0973, p-value = 0.116
## alternative hypothesis: two.sided
```

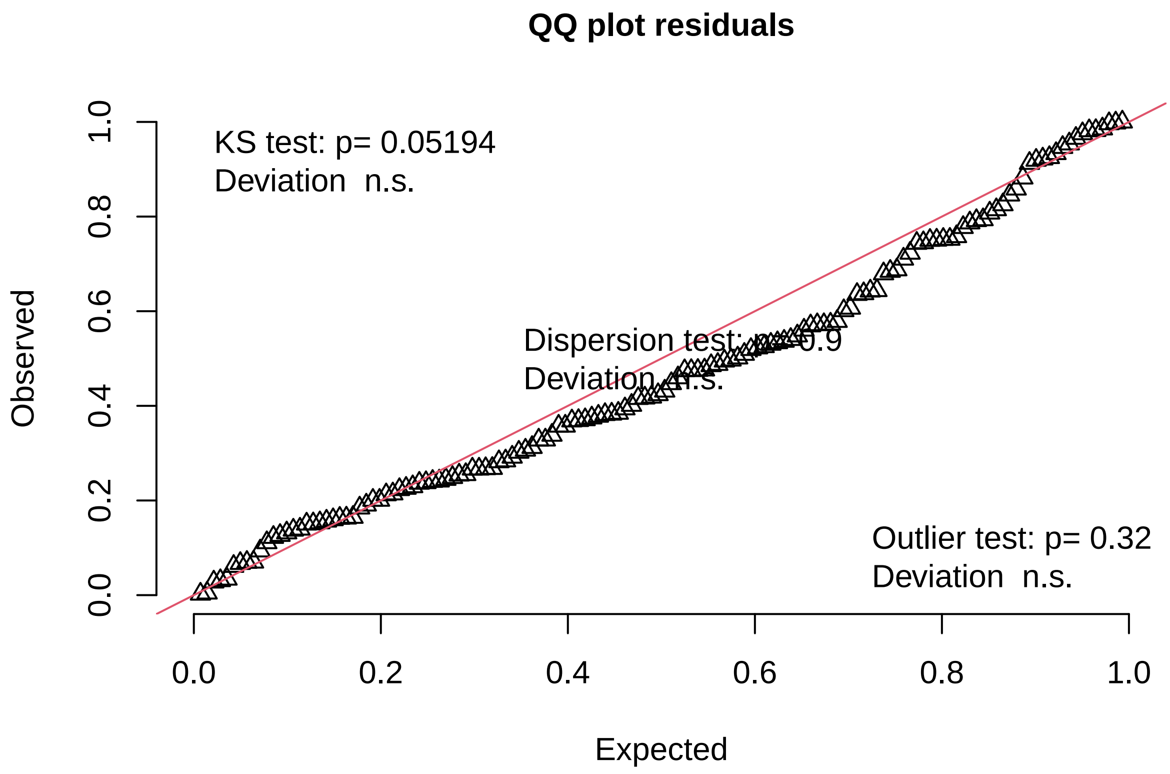


```
##
##  Asymptotic one-sample Kolmogorov-Smirnov test
##
## data:  simulationOutput$scaledResiduals
## D = 0.11419, p-value = 0.05194
## alternative hypothesis: two-sided
```

A poisson model on the number of solves, with two 2-way interaction terms between complexity & observations as well as complexity & experience fits the data well, and is a better fitting model than models with only main-effects or other combinations of interaction terms. The solve number and solve rate models are identical in fit.

Round 2 Reviewer Comment 17 - model averaging

```
# Model selection
out.put = model.sel(m1int3w,m1int42,m1int32,m1int22,m1int12,m1int3,m1int2,m1int1,m1)
out.put
```

```
## Model selection table
##          (Int) cmp Exp scl(Obs) cmp:Exp cmp:scl(Obs) Exp:scl(Obs)
## m1int32 -4.387   +   +  0.14000       +            +
## m1int3w -4.512   +   +  0.74820       +            +            +
## m1int3  -3.642   +   +  0.21430                    +
```

```
## m1int42 -4.393   +   +  0.02273       +            +            +
## m1int22 -3.644   +   +  0.22030                    +            +
## m1int2  -4.501   +   +  0.76930       +
## m1      -3.778   +   +  0.76920
## m1int12 -4.501   +   +  0.77030       +                         +
## m1int1  -3.778   +   +  0.77030                                 +
##         cmp:Exp:scl(Obs)      control df   logLik  AICc delta weight
## m1int32                                8 -124.404 265.9  0.00  0.413
## m1int3w                + g(b,l(2+05)) 10 -122.734 267.2  1.27  0.219
## m1int3                   g(b,l(2+05))  7 -126.418 267.7  1.78  0.170
## m1int42                  g(b,l(2+05))  9 -124.326 268.0  2.13  0.142
## m1int22                                8 -126.417 269.9  4.03  0.055
## m1int2                                 7 -133.360 281.6 15.66  0.000
## m1                                     6 -135.085 282.8 16.89  0.000
## m1int12                  g(b,l(2+05))  8 -133.360 283.8 17.91  0.000
## m1int1                                 7 -135.085 285.0 19.11  0.000
## Abbreviations:
##  control: g(b,l(2+05)) = 'glmerControl(bobyqa,list(2e+05))'
## Models ranked by AICc(x)
## Random terms (all models):
##   1 | Group, 1 | ID
```

```r
# model m1int32 is the best fit model with AIC weight of 0.413

# Model averaging
summary(model.avg(out.put, subset = delta <= 2))
```

```
##
## Call:
## model.avg(object = out.put, subset = delta <= 2)
##
## Component model call:
## glmer(formula = solves ~ <3 unique rhs>, data = p12, family = poisson,
##      offset = log(offset), control = <2 unique values>)
##
## Component models:
##         df  logLik   AICc delta weight
## 12345    8 -124.40 265.91  0.00   0.51
## 1234567 10 -122.73 267.17  1.27   0.27
## 1235     7 -126.42 267.68  1.78   0.21
##
## Term codes:
##                               complexity
##                                        1
##                              Experienced
##                                        2
##                      scale(Observations)
##                                        3
##                   complexity:Experienced
##                                        4
##           complexity:scale(Observations)
##                                        5
##          Experienced:scale(Observations)
##                                        6
```

```
## complexity:Experienced:scale(Observations)
##                                          7
##
## Model-averaged coefficients:
## (full average)
##                                                      Estimate Std. Error
## (Intercept)                                           -4.2634     0.7228
## complexitysimple                                       3.2043     0.8057
## scale(Observations)                                    0.3220     0.4524
## ExperiencedYes                                         1.9881     0.7977
## complexitysimple:scale(Observations)                   0.6418     0.4780
## complexitysimple:ExperiencedYes                       -1.1412     0.8898
## ExperiencedYes:scale(Observations)                    -0.1917     0.4532
## complexitysimple:ExperiencedYes:scale(Observations)    0.2855     0.5449
##                                                      Adjusted SE z value
## (Intercept)                                               0.7282   5.855
## complexitysimple                                          0.8102   3.955
## scale(Observations)                                       0.4552   0.707
## ExperiencedYes                                            0.8036   2.474
## complexitysimple:scale(Observations)                      0.4800   1.337
## complexitysimple:ExperiencedYes                           0.8942   1.276
## ExperiencedYes:scale(Observations)                        0.4554   0.421
## complexitysimple:ExperiencedYes:scale(Observations)       0.5463   0.523
##                                                      Pr(>|z|)
## (Intercept)                                           < 2e-16 ***
## complexitysimple                                     7.65e-05 ***
## scale(Observations)                                    0.4793
## ExperiencedYes                                         0.0134 *
## complexitysimple:scale(Observations)                   0.1812
## complexitysimple:ExperiencedYes                        0.2019
## ExperiencedYes:scale(Observations)                     0.6738
## complexitysimple:ExperiencedYes:scale(Observations)    0.6013
##
## (conditional average)
##                                                      Estimate Std. Error
## (Intercept)                                           -4.2634     0.7228
## complexitysimple                                       3.2043     0.8057
## scale(Observations)                                    0.3220     0.4524
## ExperiencedYes                                         1.9881     0.7977
## complexitysimple:scale(Observations)                   0.6418     0.4780
## complexitysimple:ExperiencedYes                       -1.4479     0.7485
## ExperiencedYes:scale(Observations)                    -0.7013     0.6276
## complexitysimple:ExperiencedYes:scale(Observations)    1.0442     0.5420
##                                                      Adjusted SE z value
## (Intercept)                                               0.7282   5.855
## complexitysimple                                          0.8102   3.955
## scale(Observations)                                       0.4552   0.707
## ExperiencedYes                                            0.8036   2.474
## complexitysimple:scale(Observations)                      0.4800   1.337
## complexitysimple:ExperiencedYes                           0.7552   1.917
## ExperiencedYes:scale(Observations)                        0.6335   1.107
## complexitysimple:ExperiencedYes:scale(Observations)       0.5471   1.909
##                                                      Pr(>|z|)
## (Intercept)                                           < 2e-16 ***
```

```
## complexitysimple                                        7.65e-05 ***
## scale(Observations)                                        0.4793
## ExperiencedYes                                             0.0134 *
## complexitysimple:scale(Observations)                       0.1812
## complexitysimple:ExperiencedYes                            0.0552 .
## ExperiencedYes:scale(Observations)                         0.2683
## complexitysimple:ExperiencedYes:scale(Observations)        0.0563 .
## ---
## Signif. codes:  0 '***' 0.001 '**' 0.01 '*' 0.05 '.' 0.1 ' ' 1
```

P1/P2 SOLVES results

```
m1int32 = glmer(solves ~ complexity*scale(Observations) + complexity*Experienced +
                  (1|Group) + (1|ID), family = "poisson",
                data = p12, offset=log(offset))
summary(m1int32)
```

```
## Generalized linear mixed model fit by maximum likelihood (Laplace
##   Approximation) [glmerMod]
##  Family: poisson  ( log )
## Formula:
## solves ~ complexity * scale(Observations) + complexity * Experienced +
##     (1 | Group) + (1 | ID)
##    Data: p12
##  Offset: log(offset)
##
##      AIC      BIC   logLik deviance df.resid
##    264.8    288.3   -124.4    248.8      132
##
## Scaled residuals:
##     Min      1Q  Median      3Q     Max
## -2.0702 -0.4553 -0.3427 -0.1621  5.2149
##
## Random effects:
##  Groups Name        Variance  Std.Dev.
##  ID     (Intercept) 1.157e+00 1.0755194
##  Group  (Intercept) 1.486e-07 0.0003855
## Number of obs: 140, groups:  ID, 35; Group, 6
##
## Fixed effects:
##                                      Estimate Std. Error z value Pr(>|z|)
## (Intercept)                           -4.3870     0.6691  -6.556 5.51e-11 ***
## complexitysimple                       3.3815     0.6738   5.018 5.21e-07 ***
## scale(Observations)                    0.1400     0.3001   0.467 0.640793
## ExperiencedYes                         2.1405     0.7363   2.907 0.003647 **
## complexitysimple:scale(Observations)   0.8761     0.2473   3.543 0.000395 ***
## complexitysimple:ExperiencedYes       -1.3379     0.7179  -1.864 0.062375 .
## ---
## Signif. codes:  0 '***' 0.001 '**' 0.01 '*' 0.05 '.' 0.1 ' ' 1
##
## Correlation of Fixed Effects:
##             (Intr) cmplxt scl(O) ExprnY cm:(O)
```

```
## cmplxtysmpl -0.756
## scl(Obsrvt)  0.017 -0.096
## ExperincdYs -0.871  0.694 -0.115
## cmplxty:(O) -0.085  0.016 -0.633  0.073
## cmplxtys:EY  0.720 -0.936  0.133 -0.711 -0.129
```

```
# At the reference level for covariates (complexity=complex doors,
# Experienced =No, 0 observations)
# Significantly more simple doors are solved (when 0 observations and for naive jays)
# No main effect of Observations (for naive jays on the complex doors)
# Experienced jays solve more than naive jays (when 0 observations on the complex door)
# Complexity x Observations is significant
# Complexity x Experienced is not significant at p=0.06

#Tukey's test to look at comparisons between all factor levels
library(emmeans)
c1 = emmeans(m1int32, pairwise ~ complexity:Experienced,type="response")
plot(c1$emmeans)
```

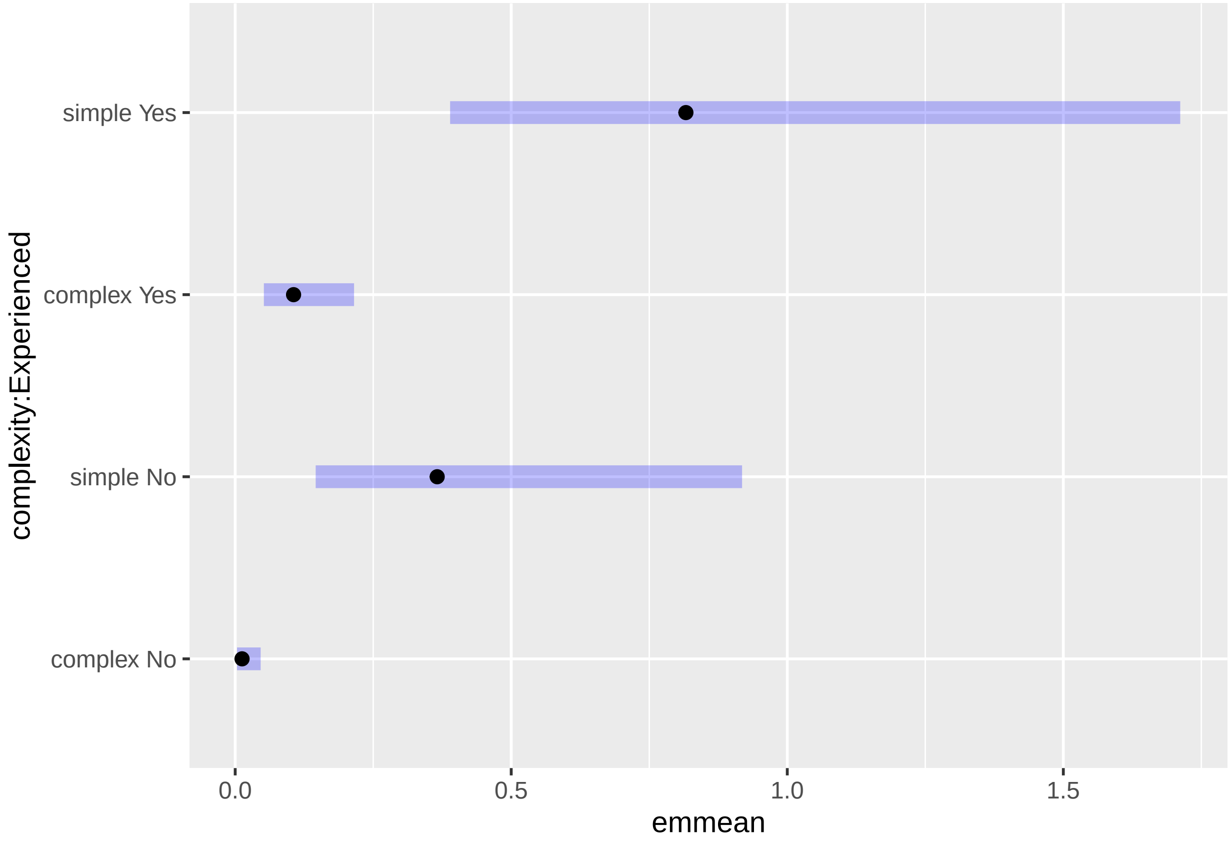


```
# As above, "Yes" refers to Experienced jays, "No" refers to naive jays.
# "Complex" refers to doors with locks, "Simple" refers to the door without the lock.
c1$contrasts %>%
     summary(infer = TRUE)
```

```
##  contrast                   ratio     SE  df asymp.LCL asymp.UCL null z.ratio
```

```
##  complex No / simple No   0.0340 0.0229 Inf   0.00602     0.192    1  -5.018
##  complex No / complex Yes 0.1176 0.0866 Inf   0.01774     0.780    1  -2.907
##  complex No / simple Yes  0.0152 0.0112 Inf   0.00229     0.101    1  -5.677
##  simple No / complex Yes  3.4592 1.9172 Inf   0.83293    14.367    1   2.239
##  simple No / simple Yes   0.4482 0.2478 Inf   0.10828     1.855    1  -1.452
##  complex Yes / simple Yes 0.1296 0.0327 Inf   0.06769     0.248    1  -8.088
##  p.value
##   <.0001
##   0.0191
##   <.0001
##   0.1128
##   0.4671
##   <.0001
##
## Confidence level used: 0.95
## Conf-level adjustment: tukey method for comparing a family of 4 estimates
## Intervals are back-transformed from the log scale
## P value adjustment: tukey method for comparing a family of 4 estimates
## Tests are performed on the log scale
```

```r
# All jays solved more at the simple door.
# Experienced jays solve more complex doors, but not simple doors, than naive jays did.


# Look at mean and variance of solves by each level
psych::describe(p12$solves[which(p12$Experienced == "Yes")])
```

```
##    vars  n mean   sd median trimmed mad min max range skew kurtosis   se
## X1    1 72 1.18 2.75      0    0.47   0   0  15    15  2.9     9.19 0.32
```

```r
psych::describe(p12$solves[which(p12$Experienced == "No")])
```

```
##    vars  n mean   sd median trimmed mad min max range skew kurtosis   se
## X1    1 68 0.24 0.69      0    0.07   0   0   4     4 3.63    14.25 0.08
```

```r
psych::describe(p12$solves[which(p12$complexity == "simple")])
```

```
##    vars  n mean   sd median trimmed  mad min max range skew kurtosis  se
## X1    1 35 1.77 2.98      1    1.21 1.48   0  15    15 2.67     8.51 0.5
```

```r
psych::describe(p12$solves[which(p12$complexity == "complex")])
```

```
##    vars   n mean   sd median trimmed mad min max range skew kurtosis   se
## X1    1 105 0.37 1.54      0       0   0   0  11    11 4.96    26.05 0.15
```

```r
# Create a figure from the model's predicted values to visualize
# the significant interaction effects
# use expand.grid to create a data frame with every combination
# of the explanatory variables from our model
# use bootstrapping to compute confidence intervals
```

```r
p12s.plot = expand.grid('Experienced' = factor(c('No', 'Yes')),
                        'Observations' = seq(0,27,1),
                        'Group' = c('CO','KI','HI','UC','XMO','TK'),
                        'complexity' = c('complex','simple')
                        ) %>%
  mutate(Experienced = factor(Experienced, levels=c('No', 'Yes')),
         complexity = factor(complexity, levels=c('complex','simple')))

mm = model.matrix(~complexity*scale(Observations) + complexity*Experienced, p12s.plot)
p12s.plot$predicted = exp(mm%*%fixef(m1int32))

predFun = function(.) mm%*% fixef(.)
# This bootstrapping function takes ~5 minutes to run
bb = bootMer(m1int32, FUN=predFun, verbose = F, nsim=200)
p12s.plot$predLCL = exp(apply(bb$t, 2, function(x) quantile(x, probs=0.025,na.rm=T)))
p12s.plot$predUCL = exp(apply(bb$t, 2, function(x) quantile(x, probs=0.975,na.rm=T)))


plot.m1 = ggplot(p12s.plot,aes(x=Observations, y=predicted[,1],
                color=Experienced, shape = complexity))+
  geom_point(size = 7, position=position_dodge(0.2), alpha = 0.6)+
  geom_line(linewidth = 0.8)+
  geom_errorbar(aes(ymin=predLCL, ymax=predUCL), width = 0, size = 0.2, alpha = 0.3,
                position=position_dodge(0.2))+
  theme_classic() +
  theme(legend.title = element_text(size = 22),
        legend.text = element_text(size = 18),
        axis.title = element_text(size = 22),
        axis.text = element_text( size = 18),
        strip.text = element_text(size = 24,face = "bold"),
        legend.justification = "left")+
  scale_x_continuous(limits=c(-0.5,28),
                     breaks=c(0,5,10,15,20,25))+
  scale_y_continuous(limits=c(-0.5,5),
                     breaks=c(0,1.0,2.0,3.0,4.0,5.0),
                     oob = squish
                     )+
  scale_color_manual(values=c("grey","#414141")) +
  guides(color=guide_legend(title="Experienced?"),
         shape=guide_legend(title="Door type")) +
  xlab("Number of observed interactions") + ylab("Predicted solves")
```

P1/P2 ATTEMPTS model selection

```
##       chisq       ratio         rdf           p
##  98.1934147   0.7327867 134.0000000   0.9912918
```

```
## Data: p12
## Models:
## m2: attempts ~ complexity + Experienced + scale(Observations) + (1 | Group) + (1 | ID)
## m2int1: attempts ~ complexity + scale(Observations) * Experienced + (1 | Group) + (1 | ID)
##        npar    AIC    BIC  logLik deviance  Chisq Df Pr(>Chisq)
```

```
## m2        6 351.66 369.31 -169.83   339.66
## m2int1    7 351.72 372.32 -168.86   337.72 1.9329  1     0.1644

## Data: p12
## Models:
## m2: attempts ~ complexity + Experienced + scale(Observations) + (1 | Group) + (1 | ID)
## m2int2: attempts ~ complexity * Experienced + scale(Observations) + (1 | Group) + (1 | ID)
##        npar    AIC    BIC  logLik deviance  Chisq Df Pr(>Chisq)
## m2        6 351.66 369.31 -169.83   339.66
## m2int2    7 352.98 373.57 -169.49   338.98 0.6796  1     0.4097

## Data: p12
## Models:
## m2: attempts ~ complexity + Experienced + scale(Observations) + (1 | Group) + (1 | ID)
## m2int3: attempts ~ Experienced + scale(Observations) * complexity + (1 | Group) + (1 | ID)
##        npar    AIC    BIC  logLik deviance  Chisq Df Pr(>Chisq)
## m2        6 351.66 369.31 -169.83   339.66
## m2int3    7 352.62 373.21 -169.31   338.62 1.0341  1     0.3092

## Data: p12
## Models:
## m2: attempts ~ complexity + Experienced + scale(Observations) + (1 | Group) + (1 | ID)
## m2int12: attempts ~ complexity * Experienced + scale(Observations) * Experienced + (1 | Group) + (1
##         npar    AIC    BIC  logLik deviance  Chisq Df Pr(>Chisq)
## m2         6 351.66 369.31 -169.83   339.66
## m2int12    8 353.04 376.58 -168.52   337.04 2.6125  2     0.2708

## Data: p12
## Models:
## m2: attempts ~ complexity + Experienced + scale(Observations) + (1 | Group) + (1 | ID)
## m2int22: attempts ~ complexity * scale(Observations) + scale(Observations) * Experienced + (1 | Grou
##         npar    AIC    BIC  logLik deviance  Chisq Df Pr(>Chisq)
## m2         6 351.66 369.31 -169.83   339.66
## m2int22    8 352.69 376.22 -168.34   336.69 2.9663  2     0.2269

## Data: p12
## Models:
## m2: attempts ~ complexity + Experienced + scale(Observations) + (1 | Group) + (1 | ID)
## m2int32: attempts ~ complexity * scale(Observations) + complexity * Experienced + (1 | Group) + (1 |
##         npar    AIC    BIC  logLik deviance  Chisq Df Pr(>Chisq)
## m2         6 351.66 369.31 -169.83   339.66
## m2int32    8 354.23 377.76 -169.12   338.23 1.4279  2     0.4897

## Data: p12
## Models:
## m2: attempts ~ complexity + Experienced + scale(Observations) + (1 | Group) + (1 | ID)
## m2int42: attempts ~ complexity * Experienced + scale(Observations) * Experienced + complexity * scal
##         npar    AIC    BIC  logLik deviance  Chisq Df Pr(>Chisq)
## m2         6 351.66 369.31 -169.83   339.66
## m2int42    9 354.33 380.80 -168.16   336.33 3.3299  3     0.3435

## Data: p12
```

```
## Models:
## m2: attempts ~ complexity + Experienced + scale(Observations) + (1 | Group) + (1 | ID)
## m2int3w: attempts ~ complexity * Experienced * scale(Observations) + (1 | Group) + (1 | ID)
##         npar    AIC    BIC  logLik deviance  Chisq Df Pr(>Chisq)
## m2         6 351.66 369.31 -169.83   339.66
## m2int3w   10 353.59 383.00 -166.79   333.59 6.0689  4     0.1941
```

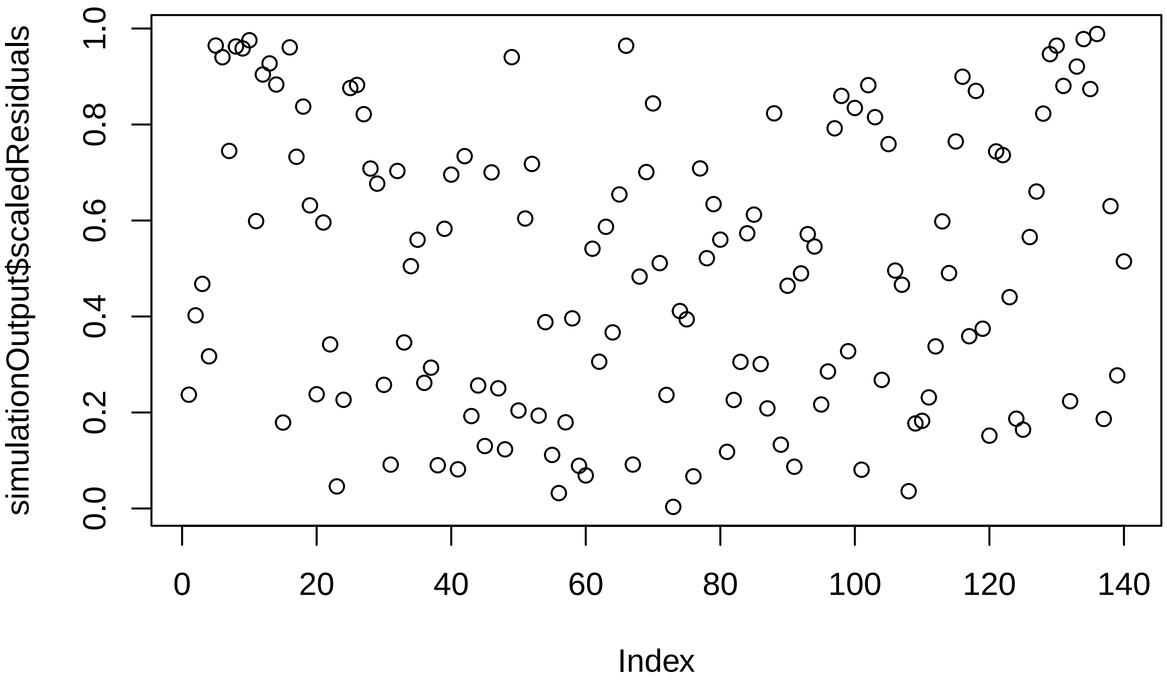

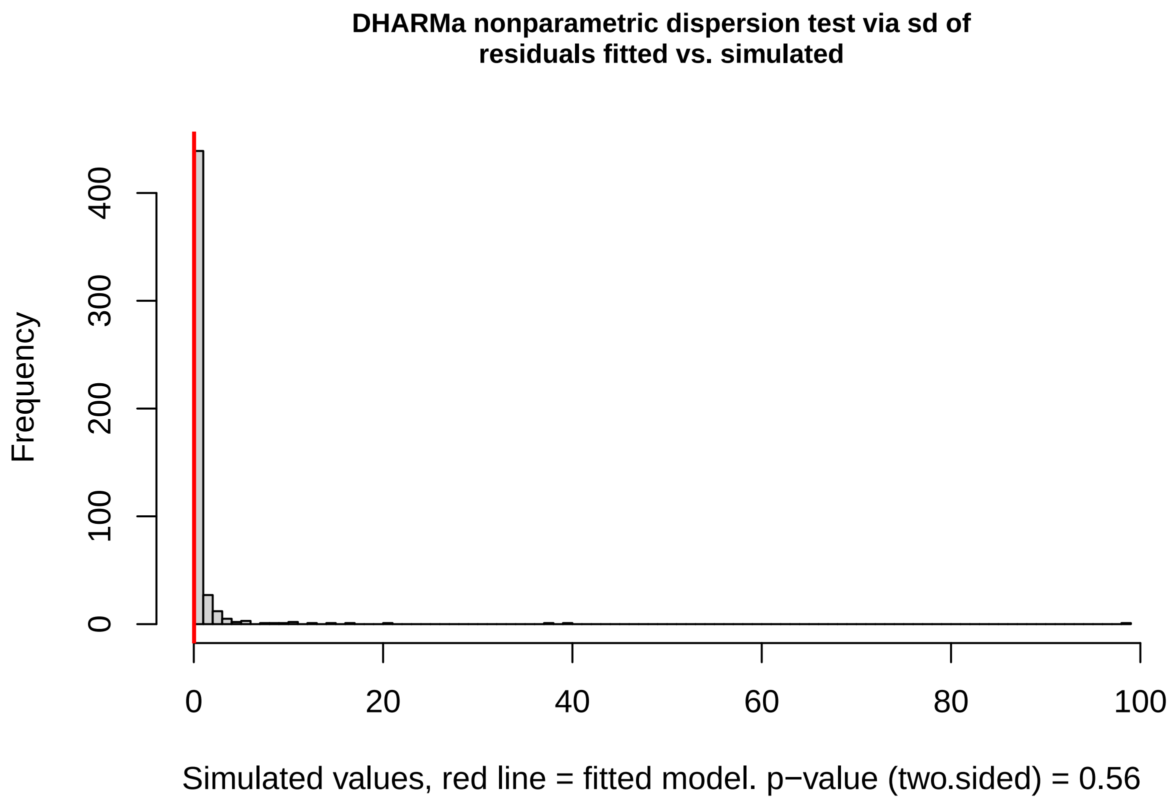


```
##
##  DHARMa nonparametric dispersion test via sd of residuals fitted vs.
##  simulated
##
## data:  simulationOutput
## dispersion = 0.034788, p-value = 0.56
## alternative hypothesis: two.sided
```

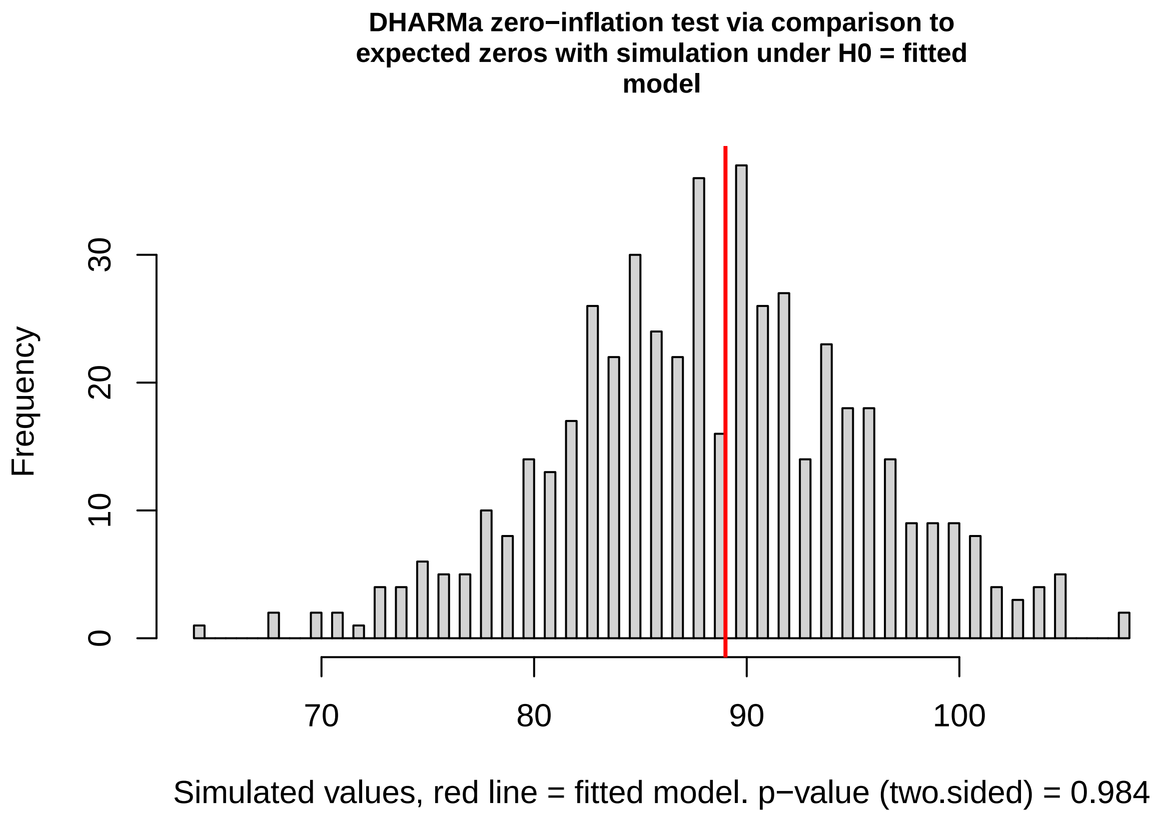


```
##
##  DHARMa zero-inflation test via comparison to expected zeros with
##  simulation under H0 = fitted model
##
## data:  simulationOutput
## ratioObsSim = 1.0055, p-value = 0.984
## alternative hypothesis: two.sided
```

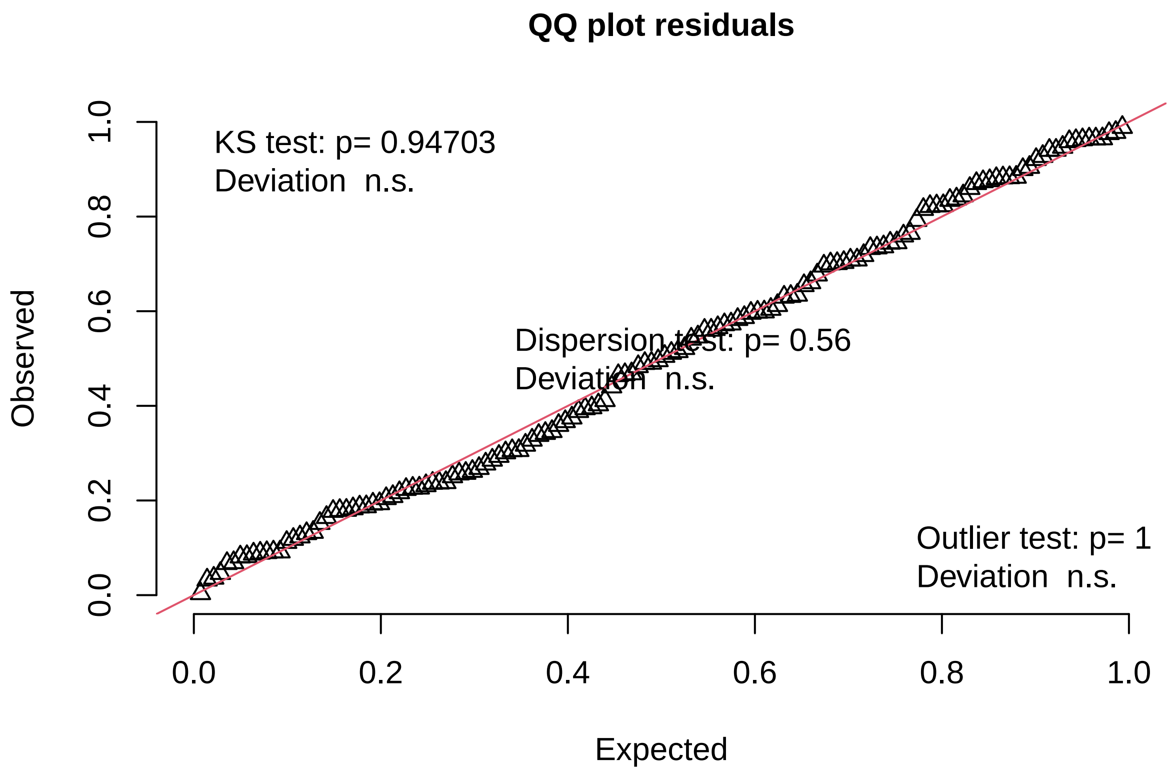


```
##
##  Asymptotic one-sample Kolmogorov-Smirnov test
##
## data:  simulationOutput$scaledResiduals
## D = 0.04423, p-value = 0.947
## alternative hypothesis: two-sided
```

A poisson model on the number of attempts, with only main effects fits the data well, and fit is not improved with models that include 1-way, 2-way or 3-way interaction terms. The attempt number and attempt rate models are identical in fit.

Round 2 Reviewer Comment 17 - model averaging

```
# Model selection
out.put = model.sel(m2int3w,m2int42,m2int32,m2int22,m2int12,m2int3,m2int2,m2int1,m2)
out.put
```

```
## Model selection table
##           (Int) cmp Exp scl(Obs) cmp:Exp cmp:scl(Obs) Exp:scl(Obs)
## m2       -2.476   +   +   0.9367
## m2int1   -2.487   +   +   1.3760                                 +
## m2int3   -2.494   +   +   0.9584                    +
## m2int22 -2.504   +   +   1.3980                    +             +
```

```
## m2int2  -2.453   +   +   0.9367       +
## m2int12 -2.464   +   +   1.3760       +                          +
## m2int3w -2.458   +   +   1.3620       +            +             +
## m2int32 -2.468   +   +   0.9545       +            +
## m2int42 -2.480   +   +   1.3910       +            +             +
##         cmp:Exp:scl(Obs)      control df   logLik  AICc delta weight
## m2                                     6 -169.829 352.3  0.00  0.229
## m2int1                                 7 -168.862 352.6  0.28  0.198
## m2int3                   g(b,l(2+05))  7 -169.311 353.5  1.18  0.127
## m2int22                                8 -168.345 353.8  1.50  0.108
## m2int2                                 7 -169.489 353.8  1.54  0.106
## m2int12                                8 -168.522 354.1  1.86  0.090
## m2int3w                + g(b,l(2+05)) 10 -166.794 355.3  3.00  0.051
## m2int32                                8 -169.115 355.3  3.04  0.050
## m2int42                  g(b,l(2+05))  9 -168.164 355.7  3.42  0.041
## Abbreviations:
##  control: g(b,l(2+05)) = 'glmerControl(bobyqa,list(2e+05))'
## Models ranked by AICc(x)
## Random terms (all models):
##   1 | Group, 1 | ID
```

```r
# model m2 is the best fit model with AIC weight of 0.229

# Model averaging
summary(model.avg(out.put, subset = delta <= 2))
```

```
##
## Call:
## model.avg(object = out.put, subset = delta <= 2)
##
## Component model call:
## glmer(formula = attempts ~ <6 unique rhs>, data = p12, family =
##      poisson, offset = log(offset), control = <2 unique values>)
##
## Component models:
##       df  logLik   AICc delta weight
## 123    6 -169.83 352.29  0.00   0.27
## 1236   7 -168.86 352.57  0.28   0.23
## 1235   7 -169.31 353.47  1.18   0.15
## 12356  8 -168.35 353.79  1.50   0.13
## 1234   7 -169.49 353.83  1.54   0.12
## 12346  8 -168.52 354.14  1.86   0.11
##
## Term codes:
##                      complexity                     Experienced
##                               1                               2
##             scale(Observations)          complexity:Experienced
##                               3                               4
##  complexity:scale(Observations) Experienced:scale(Observations)
##                               5                               6
##
## Model-averaged coefficients:
## (full average)
##                                     Estimate Std. Error Adjusted SE z value
```

```
## (Intercept)                          -2.48067    0.54434     0.54933   4.516
## complexitysimple                     -0.40932    0.43120     0.43436   0.942
## ExperiencedYes                        0.95087    0.64935     0.65526   1.451
## scale(Observations)                   1.14588    0.43828     0.44130   2.597
## ExperiencedYes:scale(Observations)   -0.37082    0.56013     0.56267   0.659
## complexitysimple:scale(Observations) -0.08966    0.23269     0.23399   0.383
## complexitysimple:ExperiencedYes       0.12308    0.39535     0.39780   0.309
##                                      Pr(>|z|)
## (Intercept)                           6.3e-06 ***
## complexitysimple                      0.34601
## ExperiencedYes                        0.14674
## scale(Observations)                   0.00942 **
## ExperiencedYes:scale(Observations)    0.50987
## complexitysimple:scale(Observations)  0.70160
## complexitysimple:ExperiencedYes       0.75703
##
## (conditional average)
##                                      Estimate Std. Error Adjusted SE z value
## (Intercept)                           -2.4807     0.5443      0.5493   4.516
## complexitysimple                      -0.4093     0.4312      0.4344   0.942
## ExperiencedYes                         0.9509     0.6494      0.6553   1.451
## scale(Observations)                    1.1459     0.4383      0.4413   2.597
## ExperiencedYes:scale(Observations)    -0.8019     0.5769      0.5822   1.377
## complexitysimple:scale(Observations)  -0.3280     0.3463      0.3495   0.939
## complexitysimple:ExperiencedYes        0.5375     0.6781      0.6844   0.785
##                                      Pr(>|z|)
## (Intercept)                           6.3e-06 ***
## complexitysimple                      0.34601
## ExperiencedYes                        0.14674
## scale(Observations)                   0.00942 **
## ExperiencedYes:scale(Observations)    0.16839
## complexitysimple:scale(Observations)  0.34792
## complexitysimple:ExperiencedYes       0.43220
## ---
## Signif. codes:  0 '***' 0.001 '**' 0.01 '*' 0.05 '.' 0.1 ' ' 1
```

P1/P2 ATTEMPTS results

```
m2 = glmer(attempts ~ complexity + scale(Observations) + Experienced +
             (1|Group) + (1|ID), family = "poisson",
           data = p12, offset = log(offset)
           ,control=glmerControl(optimizer="bobyqa",optCtrl=list(maxfun=2e5)))
summary(m2)
```

```
## Generalized linear mixed model fit by maximum likelihood (Laplace
##   Approximation) [glmerMod]
##  Family: poisson  ( log )
## Formula: attempts ~ complexity + scale(Observations) + Experienced + (1 |
##     Group) + (1 | ID)
##    Data: p12
##  Offset: log(offset)
## Control: glmerControl(optimizer = "bobyqa", optCtrl = list(maxfun = 2e+05))
```

```
##
##      AIC      BIC   logLik deviance df.resid
##    351.7    369.3   -169.8    339.7      134
##
## Scaled residuals:
##     Min      1Q  Median      3Q     Max
## -2.6810 -0.4308 -0.2725  0.1562  2.7344
##
## Random effects:
##  Groups Name        Variance Std.Dev.
##  ID     (Intercept) 2.476    1.573
##  Group  (Intercept) 0.000    0.000
## Number of obs: 140, groups:  ID, 35; Group, 6
##
## Fixed effects:
##                     Estimate Std. Error z value Pr(>|z|)
## (Intercept)          -2.4762     0.5486  -4.513 6.38e-06 ***
## complexitysimple     -0.3618     0.2879  -1.257  0.20884
## scale(Observations)   0.9367     0.3053   3.068  0.00216 **
## ExperiencedYes        0.9043     0.6506   1.390  0.16457
## ---
## Signif. codes:  0 '***' 0.001 '**' 0.01 '*' 0.05 '.' 0.1 ' ' 1
##
## Correlation of Fixed Effects:
##             (Intr) cmplxt scl(O)
## cmplxtysmpl -0.038
## scl(Obsrvt) -0.189  0.000
## ExperincdYs -0.734  0.000 -0.004
## optimizer (bobyqa) convergence code: 0 (OK)
## boundary (singular) fit: see help('isSingular')
```

```r
# No difference in attempts based on door complexity
# No difference in attempts based on jay experience
# Attempts increase as observations increase

psych::describe(p12$attempts[which(p12$Experienced == "Yes")])
```

```
##    vars  n mean   sd median trimmed mad min max range skew kurtosis   se
## X1    1 72 1.68 3.09      0    0.97   0   0  18    18 2.97    10.59 0.36
```

```r
psych::describe(p12$attempts[which(p12$Experienced == "No")])
```

```
##    vars  n mean   sd median trimmed mad min max range skew kurtosis   se
## X1    1 68 0.88 2.12      0    0.34   0   0  11    11 2.95     8.75 0.26
```

```r
psych::describe(p12$attempts[which(p12$complexity == "simple")])
```

```
##    vars  n mean   sd median trimmed mad min max range skew kurtosis   se
## X1    1 36 0.39 0.96      0    0.17   0   0   4     4 2.91     7.97 0.16
```

```r
psych::describe(p12$attempts[which(p12$complexity == "complex")])
```

```
##    vars   n mean   sd median trimmed mad min max range skew kurtosis   se
## X1    1 105  1.6 2.99      0    0.88   0   0  18    18 2.78     9.24 0.29
```

```r
p12a.plot = expand.grid('Experienced' = factor(c('No', 'Yes')),
                        'Observations' = seq(0,27,1),
                        'Group' = c('CO','KI','HI','UC','XMO','TK'),
                        'complexity' = c('complex','simple')) %>%
  mutate(Experienced = factor(Experienced, levels=c('No', 'Yes')),
         complexity = factor(complexity, levels=c('complex','simple')))

mm = model.matrix(~complexity+Experienced+scale(Observations), p12a.plot)
p12a.plot$predicted = exp(mm%*%fixef(m2))

predFun = function(.) mm%*% fixef(.)
bb = bootMer(m2, FUN=predFun, verbose = F, nsim=200)
p12a.plot$predLCL = exp(apply(bb$t, 2, function(x) quantile(x, probs=0.025,na.rm=T)))
p12a.plot$predUCL = exp(apply(bb$t, 2, function(x) quantile(x, probs=0.975,na.rm=T)))


plot.m2 = ggplot(p12a.plot,aes(x=Observations, y=predicted,
                               color=Experienced, shape = complexity))+
  geom_point(size = 7, position=position_dodge(0.2), alpha = 0.6)+
  geom_line(linewidth = 0.8)+
  geom_errorbar(aes(ymin=predLCL, ymax=predUCL), width = 0, size = 0.2, alpha = 0.3,
                position=position_dodge(0.2))+
  theme_classic() +
  theme(legend.title = element_text(size = 22),
        legend.text = element_text(size = 18),
        axis.title = element_text(size = 22),
        axis.text = element_text( size = 18),
        strip.text = element_text(size = 24,face = "bold"),
        legend.justification = "left")+
  theme(plot.margin = margin(0.4,0,1,1, "cm"))+
  scale_x_continuous(limits=c(-0.5,28),
                     breaks=c(0,5,10,15,20,25))+
  scale_y_continuous(limits=c(-0.15,1.5),
                     breaks=c(0,0.5,1,1.5),
                     oob = squish) +
  scale_color_manual(values=c("grey","#414141")) +
  guides(color=guide_legend(title="Experienced?"),
         shape=guide_legend(title="Door type")) +
  xlab("Number of observed interactions") + ylab("Predicted attempts")
```

Figure 2

```r
knitr::opts_chunk$set(warning = FALSE, message = FALSE)
p1p2 = ggarrange(plot.m2+rremove("ylab") + rremove("xlab"),plot.m1+rremove("ylab") +
                 rremove("xlab"),
                 common.legend = T, legend = "right",
```

```r
                 labels = c("a) Attempts","b) Solves"),
                 label.y = 1, label.x = 0.2,
                 font.label=list(color="black",size=22),
                 nrow = 1,ncol = 2,align = "hv")
annotate_figure(p1p2,
                left = text_grob("Predicted values", rot = 90, size = 22),
                bottom = text_grob("Number of observed interactions", x=0.47, size=22))
```

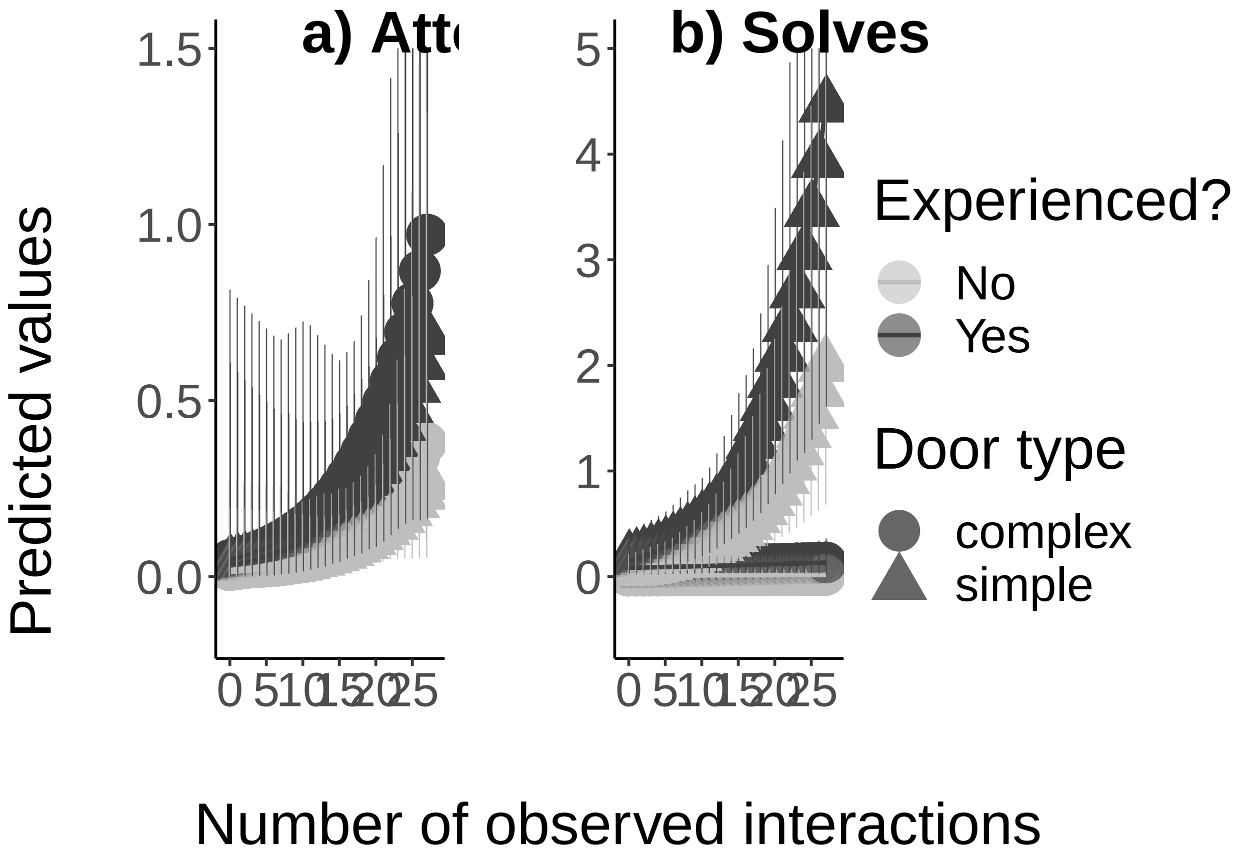

Round 1 Reviewer comment 56 - Confidence intervals for betas

```r
## Again, the Solves model is:
summary(m1int32)
```

```
## Generalized linear mixed model fit by maximum likelihood (Laplace
##   Approximation) [glmerMod]
##  Family: poisson  ( log )
## Formula:
## solves ~ complexity * scale(Observations) + complexity * Experienced +
##     (1 | Group) + (1 | ID)
##    Data: p12
##  Offset: log(offset)
##
##      AIC      BIC   logLik deviance df.resid
```

```
##    264.8    288.3   -124.4    248.8      132
##
## Scaled residuals:
##     Min      1Q  Median      3Q     Max
## -2.0702 -0.4553 -0.3427 -0.1621  5.2149
##
## Random effects:
##  Groups Name        Variance  Std.Dev.
##  ID     (Intercept) 1.157e+00 1.0755194
##  Group  (Intercept) 1.486e-07 0.0003855
## Number of obs: 140, groups:  ID, 35; Group, 6
##
## Fixed effects:
##                                        Estimate Std. Error z value Pr(>|z|)
## (Intercept)                             -4.3870     0.6691  -6.556 5.51e-11 ***
## complexitysimple                         3.3815     0.6738   5.018 5.21e-07 ***
## scale(Observations)                      0.1400     0.3001   0.467 0.640793
## ExperiencedYes                           2.1405     0.7363   2.907 0.003647 **
## complexitysimple:scale(Observations)    0.8761     0.2473   3.543 0.000395 ***
## complexitysimple:ExperiencedYes        -1.3379     0.7179  -1.864 0.062375 .
## ---
## Signif. codes:  0 '***' 0.001 '**' 0.01 '*' 0.05 '.' 0.1 ' ' 1
##
## Correlation of Fixed Effects:
##             (Intr) cmplxt scl(O) ExprnY cm:(O)
## cmplxtysmpl -0.756
## scl(Obsrvt)  0.017 -0.096
## ExperincdYs -0.871  0.694 -0.115
## cmplxty:(O) -0.085  0.016 -0.633  0.073
## cmplxtys:EY  0.720 -0.936  0.133 -0.711 -0.129
```

```
## Calculate 95% confidence intervals for each beta
#complexity ß = 3.38, se = 0.67
3.38 - 1.96*0.67 #lower
```

```
## [1] 2.0668
```

```
3.38 + 1.96*0.67 #upper
```

```
## [1] 4.6932
```

```
#experienced ß = 2.14, se = 0.74
2.14 - 1.96*0.74
```

```
## [1] 0.6896
```

```
2.14 + 1.96*0.74
```

```
## [1] 3.5904
```

```r
#observations ß = 0.14, se = 0.30
0.14 - 1.96*0.30
```

```
## [1] -0.448
```

```r
0.14 + 1.96*0.30
```

```
## [1] 0.728
```

```r
#complexity*experienced ß = -1.34, se = 0.72
-1.34 - 1.96*0.72
```

```
## [1] -2.7512
```

```r
-1.34 + 1.96*0.72
```

```
## [1] 0.0712
```

```r
#complexity*observations ß = 0.88, se = 0.25
0.88 - 1.96*0.25
```

```
## [1] 0.39
```

```r
0.88 + 1.96*0.25
```

```
## [1] 1.37
```

```r
## The Attempts model is:
summary(m2)
```

```
## Generalized linear mixed model fit by maximum likelihood (Laplace
##   Approximation) [glmerMod]
##  Family: poisson  ( log )
## Formula: attempts ~ complexity + scale(Observations) + Experienced + (1 |
##     Group) + (1 | ID)
##    Data: p12
##  Offset: log(offset)
## Control: glmerControl(optimizer = "bobyqa", optCtrl = list(maxfun = 2e+05))
##
##      AIC      BIC   logLik deviance df.resid
##    351.7    369.3   -169.8    339.7      134
##
## Scaled residuals:
##     Min      1Q  Median      3Q     Max
## -2.6810 -0.4308 -0.2725  0.1562  2.7344
##
## Random effects:
##  Groups Name        Variance Std.Dev.
##  ID     (Intercept) 2.476    1.573
##  Group  (Intercept) 0.000    0.000
```

```
## Number of obs: 140, groups:  ID, 35; Group, 6
##
## Fixed effects:
##                       Estimate Std. Error z value Pr(>|z|)
## (Intercept)            -2.4762     0.5486  -4.513 6.38e-06 ***
## complexitysimple       -0.3618     0.2879  -1.257  0.20884
## scale(Observations)     0.9367     0.3053   3.068  0.00216 **
## ExperiencedYes          0.9043     0.6506   1.390  0.16457
## ---
## Signif. codes:  0 '***' 0.001 '**' 0.01 '*' 0.05 '.' 0.1 ' ' 1
##
## Correlation of Fixed Effects:
##             (Intr) cmplxt scl(O)
## cmplxtysmpl -0.038
## scl(Obsrvt) -0.189  0.000
## ExperincdYs -0.734  0.000 -0.004
## optimizer (bobyqa) convergence code: 0 (OK)
## boundary (singular) fit: see help('isSingular')
```

```
## Calculate 95% confidence intervals for each beta
#complexity ß = -0.36, se = 0.55
-0.36 - 1.96*0.55 #lower
```

```
## [1] -1.438
```

```
-0.36 + 1.96*0.55 #upper
```

```
## [1] 0.718
```

```
#experienced ß = 0.90, se = 0.65
0.90 - 1.96*0.65
```

```
## [1] -0.374
```

```
0.90 + 1.96*0.65
```

```
## [1] 2.174
```

```
#observations ß = 0.94, se = 0.31
0.94 - 1.96*0.31
```

```
## [1] 0.3324
```

```
0.94 + 1.96*0.31
```

```
## [1] 1.5476
```

```
### Can also use confint(model object) function
```

Round 1 Reviewer comment 43 - influence of Age on performance of naive vs experienced jays

It is really difficult to know the exact age in birds if they are not caught in their first year of life. Mexican jay juveniles have white coloration on their bills until they are about 3 years of age. Using this, we were able to roughly approximate which individuals were adults (> age 3) or juveniles (age 1-3) during the experiment in 2019.

```
table(sum.data19$Experienced,sum.data19$Categorical.Age)
```

```
##
##        A  J
##   No  13  4
##   Yes 18  0
```

```
#4 (24%) of the naive jays were juveniles

# do results change if we exclude the 4 juveniles?
## Attempts
m2.adults = glmer(attempts ~ complexity + scale(Observations) + Experienced +
                    (1|Group) + (1|ID), family = "poisson",
           data = p12[-which(p12$Categorical.Age == "J"),], offset = log(offset)
           )
summary(m2.adults)
```

```
## Generalized linear mixed model fit by maximum likelihood (Laplace
##   Approximation) [glmerMod]
##  Family: poisson  ( log )
## Formula: attempts ~ complexity + scale(Observations) + Experienced + (1 |
##     Group) + (1 | ID)
##    Data: p12[-which(p12$Categorical.Age == "J"), ]
##  Offset: log(offset)
##
##      AIC      BIC   logLik deviance df.resid
##    346.3    363.2   -167.1    334.3      118
##
## Scaled residuals:
##     Min      1Q  Median      3Q     Max
## -2.6713 -0.4908 -0.3380  0.2961  2.6081
##
## Random effects:
##  Groups Name        Variance Std.Dev.
##  ID     (Intercept) 2.08     1.442
##  Group  (Intercept) 0.00     0.000
## Number of obs: 124, groups:  ID, 31; Group, 6
##
## Fixed effects:
##                     Estimate Std. Error z value Pr(>|z|)
## (Intercept)          -1.9022     0.5157  -3.689 0.000225 ***
## complexitysimple     -0.3618     0.2879  -1.257 0.208852
## scale(Observations)   0.8210     0.2925   2.807 0.004996 **
## ExperiencedYes        0.4810     0.6188   0.777 0.436990
## ---
## Signif. codes:  0 '***' 0.001 '**' 0.01 '*' 0.05 '.' 0.1 ' ' 1
```

```
##
## Correlation of Fixed Effects:
##              (Intr) cmplxt scl(O)
## cmplxtysmpl -0.040
## scl(Obsrvt) -0.163  0.000
## ExperincdYs -0.754  0.000  0.020
## optimizer (Nelder_Mead) convergence code: 0 (OK)
## boundary (singular) fit: see help('isSingular')
```

```
### qualitatively similar results

## Solves
m1int32.adults = glmer(solves ~ complexity*scale(Observations) + complexity*Experienced +
                        (1|Group) + (1|ID), family = "poisson",
                data = p12[-which(p12$Categorical.Age == "J"),], offset=log(offset)
                ,control=glmerControl(optimizer="bobyqa",optCtrl=list(maxfun=2e5)))
summary(m1int32.adults)
```

```
## Generalized linear mixed model fit by maximum likelihood (Laplace
##   Approximation) [glmerMod]
##  Family: poisson  ( log )
## Formula:
## solves ~ complexity * scale(Observations) + complexity * Experienced +
##     (1 | Group) + (1 | ID)
##    Data: p12[-which(p12$Categorical.Age == "J"), ]
##  Offset: log(offset)
## Control: glmerControl(optimizer = "bobyqa", optCtrl = list(maxfun = 2e+05))
##
##      AIC      BIC   logLik deviance df.resid
##    247.8    270.4   -115.9    231.8      116
##
## Scaled residuals:
##     Min      1Q  Median      3Q     Max
## -2.0954 -0.4839 -0.3415 -0.1882  5.1984
##
## Random effects:
##  Groups Name        Variance Std.Dev.
##  ID     (Intercept) 1.176    1.085
##  Group  (Intercept) 0.000    0.000
## Number of obs: 124, groups:  ID, 31; Group, 6
##
## Fixed effects:
##                                      Estimate Std. Error z value Pr(>|z|)
## (Intercept)                           -4.0905     0.6838  -5.982 2.21e-09 ***
## complexitysimple                       2.6411     0.7320   3.608 0.000308 ***
## scale(Observations)                    0.1767     0.3198   0.553 0.580596
## ExperiencedYes                         1.8306     0.7482   2.447 0.014416 *
## complexitysimple:scale(Observations)   0.9963     0.2670   3.732 0.000190 ***
## complexitysimple:ExperiencedYes       -0.5586     0.7625  -0.733 0.463840
## ---
## Signif. codes:  0 '***' 0.001 '**' 0.01 '*' 0.05 '.' 0.1 ' ' 1
##
## Correlation of Fixed Effects:
##              (Intr) cmplxt scl(O) ExprnY cm:(O)
```

```
## cmplxtysmpl -0.652
## scl(Obsrvt) -0.058 -0.067
## ExperincdYs -0.875  0.613 -0.012
## cmplxty:(O) -0.029 -0.105 -0.603 -0.014
## cmplxtys:EY  0.630 -0.945  0.080 -0.638  0.023
## optimizer (bobyqa) convergence code: 0 (OK)
## boundary (singular) fit: see help('isSingular')
```

```
### qualitatively similar results
```

There was no difference in our results if we excluded known juveniles from the 2019 analyses, indicating age did not significantly affect interactions with the foraging apparatus.

P3 analysis

Performance of naive jays (not present for the 2015 experiment) as a function of the number of interactions they observed group members make at the foraging task

- *Survival models account for time-dependent covariates and censored data if some individuals never attempt or solve*
- *17 naive jays in this sample*

```
### Need the raw data frame with experienced column for a survival analysis
data19 = data19.o[,-c(14:21)]
experience = unique(p12[,c(1,6)]) #reference dataframe with each individual and experience level
data19 = merge(experience, data19, all = T)
data19 = data19[-which(is.na(data19$Experienced)),]

colnames(trial.times)[1] = "Video.file"
colnames(trial.times)[2] = "Clip.duration"
tmp = trial.times[,-c(5:20)]
data19 = merge(tmp, data19, by = c("Video.file","Group","Clip.duration","Date"))

#create a column for survival analysis of whether or not a jay made an attempt in each row
data19$Attm = NA
data19$Attm[which(str_detect(data19$Behav, "attempt"))]<-1
data19$Attm[is.na(data19$Attm)]<-0

#create a column for survival analysis of whether or not a jay made a success in each row
data19$success = ifelse(data19$Result == 1,1,0)
data19$success[which(str_detect(data19$Behav, "scrounge"))]<-0

#create a column for observations
data19$observe = NA
data19$observe[which(str_detect(data19$Behav, "O"))]<-1
data19$observe[is.na(data19$observe)]<-0
### observe = 0 when jays do the behavior themselves rather than observing the behavior

# But we want to know the effect of observing group members, and that is a time-varying covariate.
# So I want to create a dataframe that has a unique row for each individual
# at each unique Time that something happened
# (i.e., every time someone sees something or does something).
```

```r
# We will have more intervals than we need for each individual.
# In other words, two subsequent intervals could have
# identical traits, but that shouldn't matter.


library(tidyverse)
data.naive = data19[which(data19$Experienced == "No"),] #remove Experienced jay data
data.naive = data.naive[-which(str_detect(data.naive$Behav, "scrounge")),] #remove scrounges

tmp = data.naive %>%
  dplyr::select(observe, Attm, success, ID, Group, Tot.end) %>%
  arrange(Group, ID, Tot.end)

#create a dataframe with the time intervals between each event
#i.e., any time an event happens where a jay did something or saw something,
#create a time interval and populate rows for each individual for that time interval.
df.tmp = expand.grid('ID' = unique(tmp$ID),
        'Time1' = unique(c(tmp$Tot.end, sum.trial.times$Clip.duration.sec.))) %>%
  arrange(ID, Time1) %>%
  rename(Time2 = Time1) %>%
  group_by(ID) %>%
  mutate(Time1 = lag(Time2)) %>%
  ungroup() %>%
  dplyr::select(ID, Time1, Time2) %>%
  mutate(Time1 = ifelse(is.na(Time1), 0, Time1)) %>%
  mutate(observe = 0, Attm = 0, success = 0)

groups = sum.data19[,c(1,3)]
df.tmp = merge(df.tmp, groups, by = "ID") #add back in social group ID

for(i in unique(df.tmp$ID)){ #for each bird
  for(j in unique(df.tmp$Time2)){ #for each interval
    tmp2 = data.naive %>% #in original data frame
      filter(ID==i & Tot.end < j & observe == 1) #find the first time the bird observed something
    df.tmp$observe[which(df.tmp$ID==i & df.tmp$Time2==j)] = nrow(tmp2)

    tmp2 = data.naive %>%
      filter(ID==i & Tot.end <= j & Attm == 1) #find the first time the bird interacted
    df.tmp$Attm[which(df.tmp$ID==i & df.tmp$Time2==j)] = nrow(tmp2)

    tmp2 = data.naive %>%
      filter(ID==i & Tot.end <= j & success == 1) #find the first time the bird succeeded
    df.tmp$success[which(df.tmp$ID==i & df.tmp$Time2==j)] = nrow(tmp2)
  }
}

df.tmp$ID = as.factor(as.character(df.tmp$ID))

# Survival analysis with only naive jays and a time-varying covariate
# of observed interactions by group members
library(coxme)
library(survival)
```

```r
## Attempts model
df.tmp.a = df.tmp[-which(df.tmp$Attm > 1),]
# only want the data up until the first attempt
df.a = df.tmp.a %>%
  arrange(ID, Time1) %>%
  group_by(ID) %>%
  mutate(testVal = lag(Attm)) %>%
  ungroup() %>%
  mutate(testVal = ifelse(is.na(testVal), 0, testVal)) %>%
  filter(testVal < 1)
# this code insures we are only looking at the time points and number of observations
# up until the jay's first attempt

Attm.fit = coxme(Surv(Time1, Time2, Attm)~ observe + (1|Group), data=df.a)
summary(Attm.fit)
```

```
## Cox mixed-effects model fit by maximum likelihood
##   Data: df.a
##   events, n = 13, 992
##   Iterations= 2 14
##                     NULL Integrated    Fitted
## Log-likelihood -30.32702  -28.95561 -28.95218
##
##                   Chisq df        p   AIC   BIC
## Integrated loglik  2.74  2 0.253750 -1.26 -2.39
##  Penalized loglik  2.75  1 0.097732  0.74  0.18
##
## Model:  Surv(Time1, Time2, Attm) ~ observe + (1 | Group)
## Fixed coefficients
##              coef exp(coef) se(coef)     z    p
## observe -1.640265 0.1939287 1.152974 -1.42 0.15
##
## Random effects
##  Group Variable  Std Dev      Variance
##  Group Intercept 0.0199972609 0.0003998904
```

```r
# p = 0.15 - No relationship between observing interactions and the latency to make an attempt
cox.zph(Attm.fit) #proportional hazards assumption not violated
```

```
##         chisq df    p
## observe  3.54  1 0.06
## GLOBAL   3.54  1 0.06
```

```r
## Calculate 95% confidence intervals for coefficient and hazard ratio
confint(Attm.fit)
```

```
##             2.5 %    97.5 %
## observe -3.900051 0.6195218
```

```r
exp(confint(Attm.fit))
```

```
##              2.5 %   97.5 %
## observe 0.02024087 1.858039
```

```r
## Solves model
df.tmp.s = df.tmp[-which(df.tmp$success > 1),]
df.s = df.tmp.s %>%
  arrange(ID, Time1) %>%
  group_by(ID) %>%
  mutate(testVal = lag(success)) %>%
  ungroup() %>%
  mutate(testVal = ifelse(is.na(testVal), 0, testVal)) %>%
  filter(testVal < 1)
# this code insures we are only looking at the time points and number of observations
# up until the jay's first success.

succ.fit = coxme(Surv(Time1, Time2, success)~observe + (1|Group), data=df.s)
summary(succ.fit)
```

```
## Cox mixed-effects model fit by maximum likelihood
##   Data: df.s
##   events, n = 8, 1463
##   Iterations= 2 14
##                       NULL Integrated    Fitted
## Log-likelihood -20.70325  -18.21273 -18.21133
##
##                    Chisq df        p  AIC  BIC
## Integrated loglik  4.98  2 0.082867 0.98 0.82
##  Penalized loglik  4.98  1 0.025664 2.98 2.90
##
## Model:  Surv(Time1, Time2, success) ~ observe + (1 | Group)
## Fixed coefficients
##              coef exp(coef)  se(coef)    z     p
## observe 0.5998524   1.82185 0.2692267 2.23 0.026
##
## Random effects
##  Group Variable  Std Dev      Variance
##  Group Intercept 0.0199927543 0.0003997102
```

```r
# p = 0.03 - a one-unit increase in observations of group members interacting increases
# the likelihood of naive jays solving by 82%
# compared to a jay that never observes interactions
cox.zph(succ.fit) #proportional hazards assumption not violated
```

```
##          chisq df   p
## observe 0.0169  1 0.9
## GLOBAL  0.0169  1 0.9
```

```r
## Calculate 95% confidence intervals for estimate and hazard ratio
confint(succ.fit)
```

```
##               2.5 %   97.5 %
## observe 0.07217772 1.127527
```

```
exp(confint(succ.fit))
```

```
##             2.5 %  97.5 %
## observe 1.074846 3.08801
```

Figure 3 - Plotting survival curves

```
attm.plot.fit = survfit(Surv(Time1, Time2, Attm)~observe, data = df.a)
attm.plot = ggsurvplot(attm.plot.fit, data = df.a, fun = 'event',
                       risk.table = F, pval = F, palette = c("black","#999999"),
                       ylab = "Proportion", size = 1.5, legend = c(0.7,0.2),
  legend.labs = c("No", "Yes"), legend.title =
    "Observed at least \n one group member \n interact at the apparatus",
  title = "a) Attempts",
  xlab = "Latency to first attempt (sec)", font.tickslab=c(10,"plain","black"),
  font.x=c(14,"plain","black"),
  font.y=c(14,"plain","black"), font.legend = c(12, "plain","black"), size=0.5,
  break.y.by = 0.25, censor=F)

df.s$bin.obs = ifelse(df.s$observe > 0,1,0)
succ.plot.fit = survfit(Surv(Time1, Time2, success)~bin.obs, data = df.s)
succ.plot = ggsurvplot(succ.plot.fit, data = df.s, fun = 'event',
                       risk.table = F, pval = F, palette = c("black","#999999"),
                       ylab = "Proportion", size = 1.5, legend = c(0.7,0.2),
  legend.labs = c("No", "Yes"), legend.title =
    "Observed at least \n one group member \n interact at the apparatus",
  title = "b) Solves",
  xlab = "Latency to first solve (sec)", font.tickslab=c(10,"plain","black"),
  font.x=c(14,"plain","black"),
  font.y=c(14,"plain","black"), font.legend = c(12, "plain","black"),
  size=0.5,ylim = c(0,1),
  break.y.by = 0.25, censor=F)

ggarrange(attm.plot$plot, succ.plot$plot, ncol =2, common.legend = T,
          legend = "right", align = "hv")
```

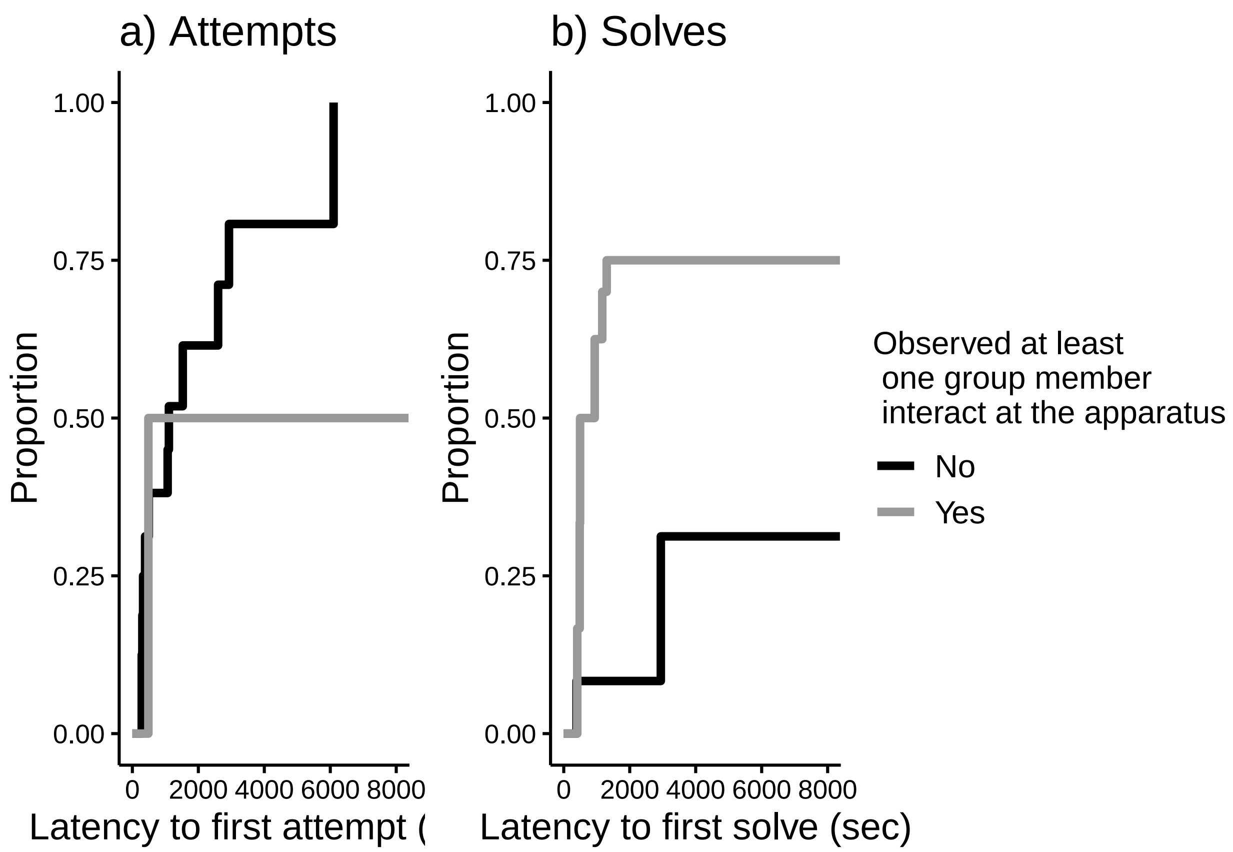

Round 1 Reviewer Comment 48 - Did demonstrators have to re-learn the task? How many attempts did demonstrators make before solving a door.

```
dem.data = data19.o[which(data19.o$ID == "XYG-GRO" |
                          data19.o$ID == "BXB-BSB" | data19.o$ID == "BYP-OWX"),]
```

We looked at how many times each of the 3 demonstrators that were still present in the population solved and attempted at the foraging task. 1 demonstrator did not interact with the task (see below). The other 2 demonstrators made very few attempts (3, max) before solving a door in 2019, indicating they were remembering how to interact with the task rather than re-learning it.

Round 2 Reviewer Comment 20 - How does demonstrator performance in 2019 compare to 2015?

```
jays15 = read.csv("Raw_Social learning dataframe.csv")
tmp = jays15[which(jays15$Individual=="XYG-GRO" & jays15$Result == 1),]
# XYG-GRO makes an accidental solve of a door it was not trained on in trial 2.
#Then first solves the door it was trained on in Trial 7 and every subsequent trial.
tmp = jays15[which(jays15$Individual=="BXB-BSB" & jays15$Result == 1),]
# BXB-BSB first solves the door it was trained on in trial 5 and in every subsequent trial.
```

Supp. Fig. 1 - change in naive jays performance before and after observing group members interact

An additional figure showing the number of solves/attempts before and after observing 1 interaction at the foraging apparatus by a group member. Unlike the survival models, above, this plot is not a test of the effect on observing group member interactions with the apparatus on the latency to first attempt or solve. Those models specifically account for the confound of time on the number of attempts and solves. This figure merely demonstrates the increase in attempts and solves after the first occurrence of an observation of a group member interacting at the task.

```
#Successes before/after observations
b4 = aggregate(success ~ ID + Group, FUN = "max",
               data = df.tmp[which(df.tmp$observe ==0),])
colnames(b4)[3]="before observations"
aft = aggregate(success ~ ID + Group, FUN = "max",
                data = df.tmp[which(df.tmp$observe > 0),])
colnames(aft)[3]="after observations"
b4aft = merge(b4,aft, by = c("ID","Group"), all = T)

su.plot = ggpaired(b4aft, cond1 = "before observations",
                   cond2 = "after observations", fill = "condition",
                   palette = c("black","grey"),
  xlab=F, ylab = "Number of solves", title = "b) Solves")

#Now attempts
b4 = aggregate(Attm ~ ID + Group, FUN = "max",
               data = df.tmp[which(df.tmp$observe ==0),])
colnames(b4)[3]="before observations"
aft = aggregate(Attm ~ ID + Group, FUN = "max",
                data = df.tmp[which(df.tmp$observe > 0),])
colnames(aft)[3]="after observations"
b4aft = merge(b4,aft, by = c("ID","Group"), all = T)

at.plot = ggpaired(b4aft, cond1 = "before observations",
                   cond2 = "after observations", fill = "condition",
                   palette = c("black","grey"),
  xlab=F, ylab = "Number of attempts",title = "a) Attempts")

ggarrange(at.plot, su.plot, ncol = 2, common.legend = T, legend = "none", align = "hv")
```

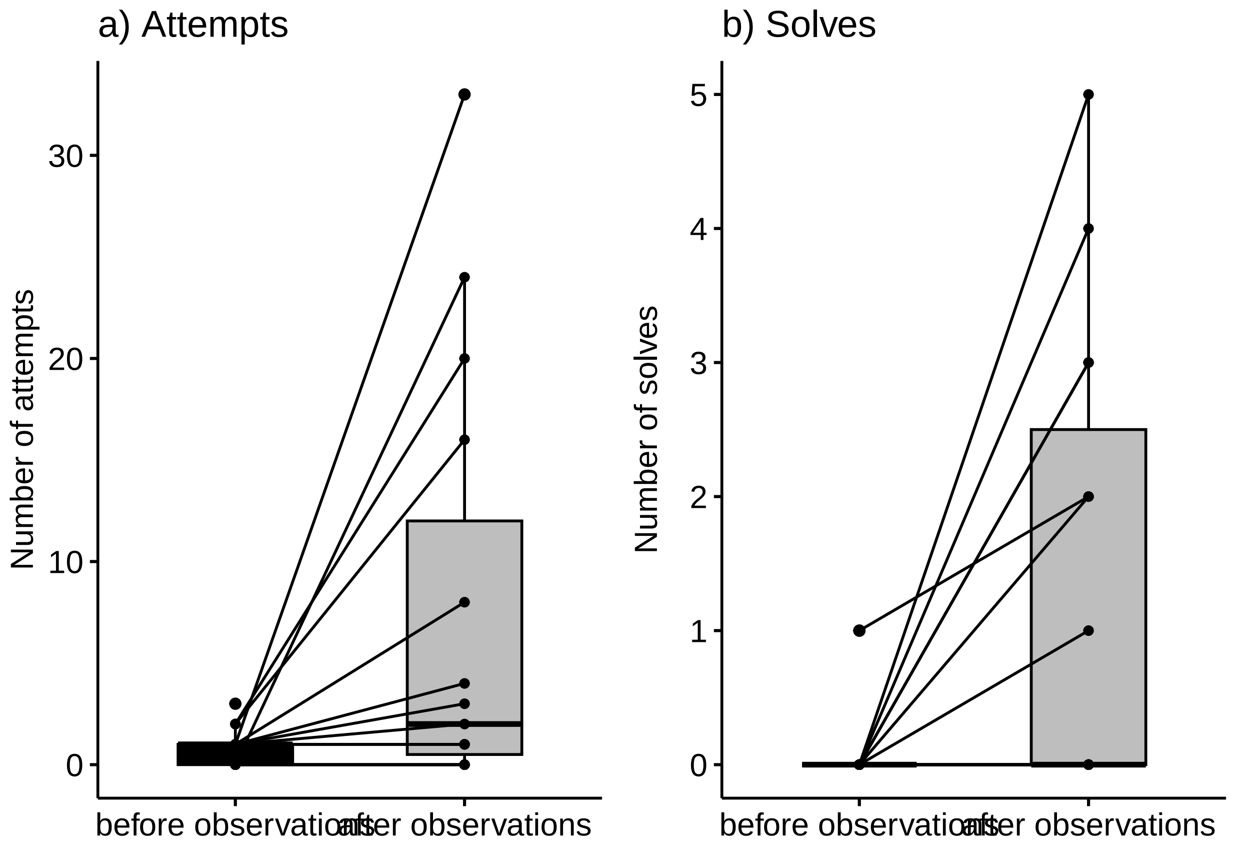

Rate models

We checked that we would get the same results if we considered the rate of attempts and solves (attempts/solves per trial time) compared to the number of attempts and solves. We found that all models yeilded similar results, so we proceeded with the attempt/solve number models.
